# Physiological and Genomic Features of a Novel Sulfur-Oxidizing Gammaproteobacterium Belonging to a Previously Uncultivated Symbiotic Lineage Isolated from a Hydrothermal Vent

**DOI:** 10.1371/journal.pone.0104959

**Published:** 2014-08-18

**Authors:** Takuro Nunoura, Yoshihiro Takaki, Hiromi Kazama, Jungo Kakuta, Shigeru Shimamura, Hiroko Makita, Miho Hirai, Masayuki Miyazaki, Ken Takai

**Affiliations:** Subsurface Geobiology & Advanced Research (SUGAR) Project, Extremobiosphere Research Program, Institute of Biogeosciences, Japan Agency for Marine-Earth Science & Technology (JAMSTEC), Yokosuka, Japan; University of Vienna, Austria

## Abstract

Strain Hiromi 1, a sulfur-oxidizing gammaproteobacterium was isolated from a hydrothermal vent chimney in the Okinawa Trough and represents a novel genus that may include a phylogenetic group found as endosymbionts of deep-sea gastropods. The SSU rRNA gene sequence similarity between strain Hiromi 1 and the gastropod endosymbionts was approximately 97%. The strain was shown to grow both chemolithoautotrophically and chemolithoheterotrophically with an energy metabolism of sulfur oxidation and O_2_ or nitrate reduction. Under chemolithoheterotrophic growth conditions, the strain utilized organic acids and proteinaceous compounds as the carbon and/or nitrogen sources but not the energy source. Various sugars did not support growth as a sole carbon source. The observation of chemolithoheterotrophy in this strain is in line with metagenomic analyses of endosymbionts suggesting the occurrence of chemolithoheterotrophy in gammaproteobacterial symbionts. Chemolithoheterotrophy and the presence of homologous genes for virulence- and quorum sensing-related functions suggest that the sulfur-oxidizing chomolithotrophic microbes seek animal bodies and microbial biofilm formation to obtain supplemental organic carbons in hydrothermal ecosystems.

## Introduction

Deep-sea hydrothermal vent ecosystems are among the most productive oceanic ecosystems on the planet and are sustained by primary production of chemolithoautotrophic microorganisms that feed on inorganic energy sources and nutrients from hydrothermal fluids. The oxidation of reduced sulfur compounds such as HS^−^, S^0^, polysulfide and thiosulfate, coupled to reduction of O_2_ and/or nitrate is one of the predominant forms of energy metabolism of chemolithoautotrophs in the hydrothermal mixing zones, where reductive hydrothermal fluids are diluted with oxygenated ambient seawater [1], [2], [3]. Among the sulfur oxidizers that have planktonic, adhesive and/or symbiotic lifestyles in hydrothermal vent ecosystems, *Gamma*- and *Epsilonproteobacteria* are recognized as the dominant populations considering their huge biomass in mixing zone of hydrothermal fluids and deep-sea water. Especially in the endosymbiotic population, most of them belong to *Gammaproteobacteria*, but a few are classified in the *Epsilonproteobacteria* [1], [3], [4].

Currently, a number of free-living diverse epsilonproteobacterial strains, some of which are phylogenetically related to the endosymbionts, have been isolated from various hydrothermal environments, and the genomic traits of a free-living strain closely related to the endosymbionts has been reported [3], [5]. In contrast, the diversity of sulfur-oxidizing isolates in *Gammaproteobacteria* from hydrothermal ecosystems is limited including the groups closely related to endosymbionts [1], [3], [4], [6], [7].

Here, we report the isolation, characterization and genomic traits of a sulfur-oxidizing facultatively chemolithoautotrophic gammaproteobacterium that is closely related to the *Ifremeria nautilei* gastropod endosymbionts. The phylogenetic group including the *I. nautilei* endosymbionts is part of a larger clade of endosymbionts in bivalves and tubeworms, for which no cultivated representative exist so far, although potential free-living phylotypes in this group have been identified in deep-sea hydrothermal environments [3], [4], [8], [9]. The physiological and genomic traits of this strain provide insights into the metabolism and ecological functions of sulfur-oxidizing *Gammaproteobacteria* in global deep-sea hydrothermal environments.

## Materials and Methods

### Ethics statement

No specific permits were required for the field study described here, and sampling locations were out of protected areas. The field study did not involve endangered or protected species.

### Sampling, enrichment, isolation, cultivation, microscopic observations and characterization

Sulfide chimney structures were obtained from vent no. 7 (28°23.29N, 127°38.37E) at the Minami-Ensei Knoll hydrothermal field in the Okinawa Trough by the *ROV Hyper Dolphin* during cruise NT07-11 (June 2007) of the *R/V Natsushima* (JAMSTEC) [10] (Kawagucci *et al*., 2013). The subsampled pieces of the sulfide structures covered by polychaete colonies from no. 7 vent in the Minami-Ensei Knoll hydrothermal field were stored anaerobically (with or without 0.05% neutralized Na_2_S) with sterilized seawater in Schott glass bottles under 100% N_2_ (100 kPa). The bottles were sealed with butyl rubber stoppers and stored at 5°C.

MMJHS medium [11] (3 ml) under a gas mixture of 80% H_2_ and 20% CO_2_ (200 kPa) in a 25 ml test tube was prepared for sulfur and/or hydrogen oxidizing bacteria and serial dilution counting of these bacteria was examined at 37, 55 and 70°C. Before obtaining a pure culture, utilization of each potential electron acceptor for hydrogen oxidation, such as thiosulfate, nitrate and nitrite [each as 0.1% (w/v) sodium salt], elemental sulfur (3% w/v), and O_2_ (1% partial pressure) was tested using MMJ medium [12] under a gas mixture of 80% H_2_ and 20% CO_2_ (200 kPa). Each of the electron acceptors associated with sulfur or thiosulfate oxidation, such as nitrate and nitrite [each as 0.1% (w/v) sodium salt] and oxygen (1% partial pressure) was examined under a gas mixture of 80% N_2_ and 20% CO_2_ (200 kPa). Based on the result of substrate utilization test, MMJSN medium (3 ml) containing S^0^ and nitrate as an electron donor and acceptor [0.3% (w/v) and 0.1% (w/v) sodium salt], respectively, under a gas mixture of 80% N_2_ and 20% CO_2_ (200 kPa) in a 25 ml test tube [13] was prepared for the isolation using the serial dilution to extinction technique [14] at 37°C.

The purity of the isolate was then tested by microscopic observation and repeated direct partial sequencing of the SSU rRNA gene with careful observation of the chromatogram as described previously [13]. Cells were routinely observed using an Olympus BX51 microscope (Tokyo, Japan).

### Microscopic observation

Growth of the isolate was determined by direct cell counting after staining using 4′,6-diamidino-phenylindole (DAPI) [15] under an Olympus BX51 epifluorescence microscope. Cells adhering to sulfur particles were released by adding carbon disulfide to dissolve elemental sulfur [16]. Cells at late exponential phase grown in MMJSN medium at 37°C were observed by both transmission and scanning electron microscope. Transmission electron micrographs of negatively stained cells and thin cell sections were obtained as described by Zillig *et al*. [17]. Scanning micrographs of cells that were attached to elemental sulfur were obtained as described previously [18].

### Physiological characterization

To determine the temperature, pH and NaCl concentration ranges for growth, cultures were grown in 3 ml MMJSN medium in 25 ml test tube under static condition using a temperature-controlled drying oven. Ranges of temperature and NaCl concentrations for the growth were 20–50°C and 0–5%, respectively. The initial pH for each medium that determined the pH range for growth (pH 5.3–8.4) was adjusted using NaHCO_3_ and Na_2_CO_3_ [0 or 0.1% (w/v)] and partial pressure of CO_2_ in the 200 kPa of gas mixture because Good buffers (MES, PIPES and HEPES) used to pH adjustment for anaerobic medium [19] inhibited the growth. The end products of sulfur oxidation coupled with nitrate reduction were examined using sulfates and NH_4_Cl-deficient MMJSN medium as described previously [13]. To determine the optimum O_2_ concentrations for growth, MMJSN medium without NaNO_3_ was used under a gas mixture in which the O_2_ concentration ranged 1.0–10.0%.

The range of sulfur compounds as electron donors, elemental sulfur (3% w/v), thiosulfate, tetrathionate [each as 0.1% (w/v) sodium salt] and sulfite [0.03% (w/v) sodium salt] was re-examined using MMJ medium with nitrate under a gas mixture of 80% N_2_: 20% CO_2_ or MMJ medium under a gas mixture of 77% N_2_: 20% CO_2_: 3% O_2_.

Nitrogen sources for growth (ammonium, nitrate, nitrite, nitrogen gas and yeast extract) were examined using MMJSN medium without nitrogen compounds, such as NH_4_Cl and NaNO_3_. Tests for utilization of nitrate, nitrite, ammonium and yeast extract, as nitrogen sources were performed under the gas mixture, 77% H_2_: 20% CO_2_: 3% O_2_ (200 kPa). Utilization of nitrogen gas was also tested under the gas mixture, 77% N_2_: 20% CO_2_: 3% O_2_, respectively (200 kPa). The concentration of inorganic nitrogen compounds as sodium or chloride salts was 0.01% (w/v) and that of yeast extract was 0.05% (w/v) in the nitrogen utilization test.

Utilization of organic carbon sources was tested using MMJSN medium without NaHCO_3_ under N_2_ atmosphere (200 kPa). Each of the following substrates were added at 0.1 or 0.01% (w/v): yeast extract (Difco), peptone (Difco), tryptone peptone (Difco), Casamino acids (Difco), gelatin, casein, starch, maltose, fructose, sucrose, lactose, galactose, cellobiose, mannose, rhamnose, xylose, mannitol, glycerol, ethanol, methanol, fumarate, tartrate, acetate, formate, citrate, pyruvate, malate, propionate, succinate, alanine, arginine, asparagine, aspartate, cystein, glutamine, glutamate, glycine, histidine, isoleucine, leucine, lysine, methionine, phenylalanine, proline, serine, threonine, tryptophan, tyrosine and valine. Effect of organic carbon sources on final cell concentration in the presence of inorganic carbon source was tested using MMJSN medium. Each of the following substrates were added at 0.1 or 0.01% (w/v); yeast extract, peptone, tryptone peptone, Casamino acids, gelatin, casein, citrate, formate, fumarate, pyruvate and succinate.

Antibiotic resistance against 25 and 100 µg ml^−1^ of erythromycin, novobiocin, tetracycline, streptomycin, chloramphenicol, vancomycin, rifampicin, kanamycin, ampicillin, spectinomycin and penicillin G was tested in MMJSN medium.

### Large-scale cultivation and genomic DNA preparation

For extraction of genomic DNA, lipid analyses and enzymatic activity measurements, cells at late exponential phase grown in 100 or 350 ml of MMJSN medium under a gas mixture of 80% N_2_ and 20% CO_2_ (100 kPa) in 250 or 1000 ml schott bottle, respectively, were obtained. Genomic DNA was prepared using the Illustra bacteria genomic Prep Mini Spin Kit (GE Healthcare).

### Analyses for fatty acid, polar lipid and quinones

The fatty acids of strain Hiromi 1 and related species were obtained from cells by saponification, methylation and extraction according to the Sherlock Microbial Identification System [20]. Fatty acid compositions were determined using a Finnigan TRACE DSQ GC-MS system (Thermo Scientific) equipped with a TR-5MS column (Thermo Scientific) under a helium flow of 1.5 ml min^−1^ and an oven temperature program was increasing from 140°C to 260°C at 4°C min^−1^ and hold 260°C for 4 min. To determine double-bond positions of unsaturated fatty acids, analyses of dimethyl disulfide derivatives were performed as described by Christie [21]. The polar lipids and isoprenoid quinones were extracted from lyophilized cells (50 mg) according to the procedures described by Minnikin *et al*. [22]. The polar lipids identified using two-dimensional TLC followed by spraying with the appropriate detection reagents [22], [23]. Isoprenoid quinones were purified on thin-layer chromatography. The purified isoprenoid quinones were analyzed using reversed-phase high-performance liquid chromatography (HPLC) [23].

### Phylogenetic analysis

The SSU rRNA gene alignment was constructed using ARB software [24]. Ambiguously aligned regions were manually edited and/or deleted. A phylogenetic tree was constructed by neighbor-joining method using Clustal X ver. 2.0 [25].

### Genome sequencing and assembly, gene identification and annotation

A mate-pair library was constructed from the genomic DNA and analyzed using the 454 GS FLX system (Roche) at Agencourt Inc (Danvers, MA, USA). A total of 3110167 bp with 237 contigs were obtained by a half plate in the FLX system, and three large scaffolds were constructed using mate-pair sequences. Unsequenced regions were obtained by PCR and analyzed by Sanger sequencing using an ABI 3730xl sequencer.

Protein-coding sequences (CDSs) were initially predicted by a combination of GeneMarkS [26] and the program Glimmer [27], and putative functions of the predicted CDSs were identified by comparing against the NCBI non-redundant (NR) database and the Kyoto Encyclopedia of Genes and Genomes (KEGG) [28] database using BLASTP [29]. Further functional information was obtained using functional domain search by the HMMER program [30] and Pfam database [31]. Genes for tRNA were identified using tRNAscan-SE [32]. Metabolic pathways were predicted by referring to the KEGG pathway and MetaCyc [33].

Sequences obtained in this study have been deposited in the DDBJ/EMBL/GenBank database under the following accession numbers: a complete chromosome of strain Hiromi 1 (AP012273) and two plasmids (AP012274 and AP012275).

### Enzymatic activity measurements and metabolomics

Autotrophically grown cells were harvested at the late exponential phase and stored in a liquid nitrogen tank until the enzymatic activity measurements were performed. The cells were resuspended in 100 mM Tris-HCl buffer (pH 7.8) and disrupted by sonication, and cell extract was obtained after centrifugation. Activities of ATP-dependent phosphofructokinase, pyrophosphate-dependent phosphofructokinase (PPi-PFK), fructose 1,6-bisphosphatase and ribulose 1,5-bisphosphate carboxylase in the cell extracts were measured at 37°C as follows.

Fructose 1,6-bisphosphate production by PPi-PFK, ATP-dependent phosphofructokinase and fructose-1,6-bisphosphatase was coupled with aldolase, glycerol phosphate dehydrogenase and triose phosphate isomerase, and NADH consumption was measured by a UV 2550 spectrophotometer (Shimadzu, Kyoto, Japan) [34], [35]. The assay mixture (1 ml) contained 100 mM HEPES-NaOH (pH 7.0), 0.25 mM NADH, 5 mM MgCl_2_, 0.5 units aldolase (Sigma), 0.5 units glycerol phosphate dehydrogenase (Sigma), triose phosphate isomerase (Sigma) and cell extract (50 µg protein). In the PPi-PFK and ATP-dependent phosphofructokinase activity measurements, 2 mM PPi and 2 mM ATP, respectively, were added in the reaction mixture. The reaction was initiated by adding 10 mM fructose 6-phosphate (Sigma). In addition, ADP production by ATP-dependent phosphofructokinase was also assessed by the coupling reaction of lactate dehydrogenase (Toyobo, Osaka, Japan) and pyruvate kinase (Sigma). The assay mixture (1 ml) contained 100 mM HEPES-NaOH (pH 7.0), 5 mM MgCl_2_, 20 mM fructose 6-phosphate, 2 mM ATP and cell extract. After 10 min of incubation at 37°C, protein-free reaction mixture possibly containing ADP was obtained using Amicon filter unit (3 kDa cutoff). Next, the ADP concentration was monitored spectrophotometrically by the consumption of NADH in the assay mixture (100 µl), which contained 100 mM Tris-HCl (pH 8.0), 5 mM MgCl_2_, 0.3 mM NADH, 0.2units lactate dehydrogenase and 0.2units pyruvate kinase.

Fructose 6-phosphate production by PPi-PFK and fructose-1,6-bisphosphatase was coupled with phosphoglucose isomerase and NADP-dependent glucose-6-phosphate dehydrogenase, and NADPH formation was measured [34], [35] (Rashid *et al*., 2002; Reshetnikov *et al*., 2008). The Assay mixture (1 ml) 100 mM HEPES-NaOH (pH 7.0), 0.4 mM NADP, 5 mM MgCl_2_, 0.5 units phosphoglucose isomerase (Sigma), 0.5 units glucose-6-phophate dehydrogenase (Sigma) and cell extract (50 µg protein). The reaction was initiated by adding 2 or 20 mM fructose 1,6-bisphosphate (Sigma), and the production of NADPH was monitored. After fructose-1,6-bisphosphatase activity was monitored, the reaction of PPi-PFK was started by adding NaH_2_PO_4_ (2 mM).

Ribulose 1,5-bisphosphate carboxylase (Rubisco) activity was measured by the production of 3-phosphoglyceraldehyde using two-step coupling reaction. The primary reaction mixture contained 10 mM Bicin/NaOH (pH 8.3), 10 mM MgCl_2_, 20 mM NaHCO_3_, and 20 mM ribulose 1,5-bisphosphate (Sigma). After ultrafiltration, the amount of 3-phosphoglyceraldehyde in the first reaction mixture was assessed by a coupling reaction. A 20 µl aliquot of the first reaction mixture was added to an 80 µl reaction mixture containing bicin/NaOH (pH 8.3), 10 mM MgCl_2_, 5 mM ATP, 0.2 mM NADH, 0.1 U of glyceraldehyde-3-phosphate dehydrogenase, 0.2 U of triosphosphate isomerase and 0.05 U of phosphoglycerate kinase (Unitika, Osaka, Japan) [36].

The extraction of metabolites and characterization of the extracted metabolites were performed using capillary electrophoresis time-of-flight mass spectrometry (CE-TOFMS) as previously described [37] with commercial electrophoresis buffer (Solution ID H3302-1021, Human Metabolome Technologies Inc., Tsuruoka, Japan) by Human Metabolome Technologies Inc.

## Results and Discussion

### Isolation and characterization of a novel strain

Growth of unique non-motile short rods with large intracellular particles was observed at 37°C from the most dilute series (10^3^ cells ml^−1^ chimney structure) showing growth in MMJHS medium under a gas mixture of 80% H_2_ and 20% CO_2_ using chimney subsamples from vent no. 7 in the Minami Ensei Knoll hydrothermal field located in the middle Okinawa Trough. Since the MMJHS medium contains several energy sources and *Epsilonprotebacteria* or *Thiomicrospira crunogena* strains have been previously enriched and isolated with this medium at 37°C [9], [11], [38], [39], various combinations of electron donors and acceptors were examined before the isolation process for the short rods. As a result, the most stable growth of the short rods was observed with sulfur oxidation coupled with nitrate reduction. Pure culture (strain Hiromi 1) was then obtained using the serial dilution to extinction technique at 37°C with MMJSN medium containing elemental sulfur and nitrate as an electron donor and acceptor, respectively.

**Figure 1:**
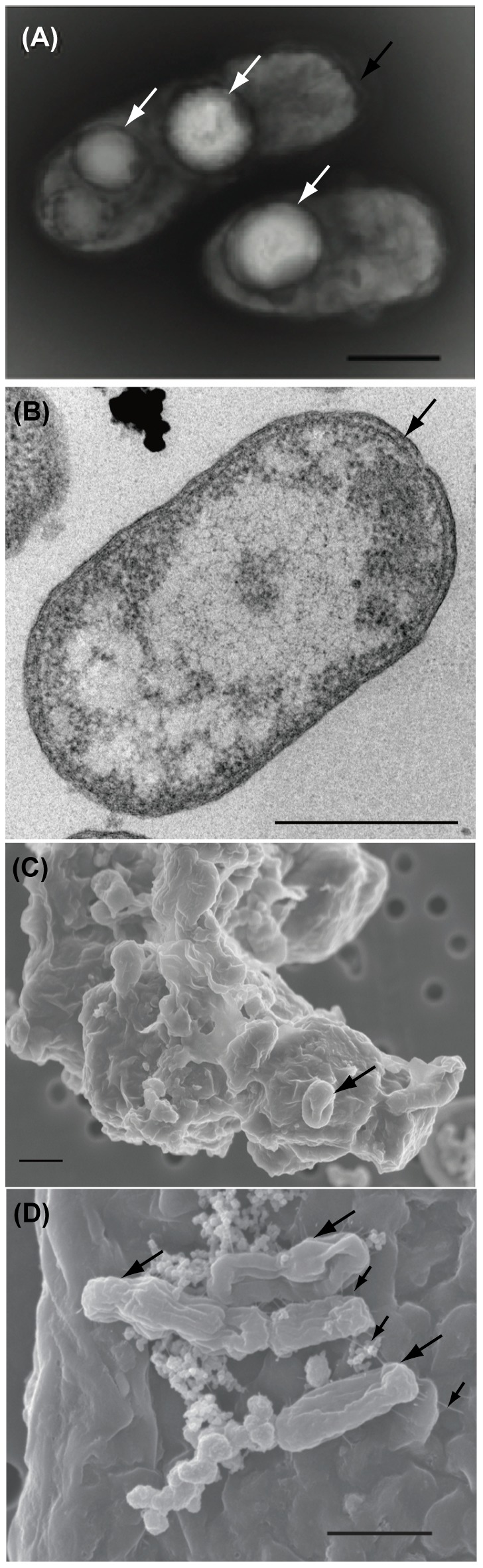
Electron micrographs of the new isolate strain Hiromi 1. A transmission electron micrograph of a negatively stained cell (A) and a thin section cell (B) grown under the chemolithoautotrophic condition. White and black arrows indicate intracellular particle and outer membrane, respectively. Scanning micrographs of cells adhering on elemental sulfur by pilus to biofilm (C, D). Cells attached on biofilm formation and pili structures were shown by large and small black arrows. Other cells grew under the polysaccharide-like substances. Bars, 0.2 µm (A), 0.5 µm (B) and 1 µm (C, D).

The non-motile cells consisted of straight short rods that were approximately 1.9 µm (1.3–4.0 µm) in length and 0.8 µm (0.45–1.2 µm) in width without a flagellum (Figure 1A). The cells had outer membrane and intracellular large particles (Fig. 1AB). The cells secreted a polysaccharide-like substance layer that covered the sulfur particles and grow under the substance layer until the space between the substance layer and sulfur particle was filled with cells (Figure 1C, D, S1). Pilus-like structures were also observed with the cells and adhered on the sulfur particle in the SEM images (Figure 1D).

Strain Hiromi 1 grew at ranges of 25–45°C, pH 5.7–8.4 and 2.0–4.5% (w/v) NaCl (Figure S2). Optimum growth was observed at 40°C, pH 6.5 and 3.0% NaCl. No growth occurred at 20 and 50°C, pH 5.3 or NaCl concentrations of 1.5 and 5.0%. Under optimum conditions grow with S^0^ and nitrate as an electron donor and acceptor, respectively, the doubling time was 3.4–4.0 hours, and the maximum cell density was 1.5×10^8^ cell ml^−1^ with elemental sulfur and nitrate as an electron donor and acceptor, respectively. Aerobic growth occurred under a head-space gas mixture (1.0–7.0% O_2_) (Figure S2). Under the optimum O_2_ concentration (2.0%) with elemental sulfur as an electron donor, the doubling time was 3.0 hours, and maximum cell density was 2.6×10^8^ cell ml^−1^.

The strain grew with sulfur, thiosulfate or tetrathionate as the sole electron donor and with oxygen or nitrate as an electron acceptor, while hydrogen and sulfite did not support growth as an electron donor. Only sulfate and nitrogen gas were detected as products of complete sulfur oxidation and nitrate reduction. Strain Hiromi 1 utilized ammonium and yeast extract but not nitrate or nitrite as nitrogen sources. N_2_ fixation did not occur. Thus, neither nitrate assimilation nor nitrite ammonification occurred.

Chemolithoheterotrophic growth of the strain was also observed with proteinaceous compounds and organic acids, such as yeast extract, peptone, tryptone peptone, casamino acids, gelatin, fumarate, formate, citrate, pyruvate and succinate coupled with sulfur oxidation in the absence of inorganic carbon. No growth was observed with sugars as a sole carbon source. The maximum final cell yield under these chemolithoheterotrophic growth conditions was approx. 2.0×10^7^ cells ml^−1^. No growth occurred with these organic substances in the absence of sulfur compounds as an electron donor. These results indicated the capability of chemolithoheterotrophic but not chemoorganoheterotrophic growth in this strain. In the presence of inorganic carbon, maximum cell yield increased to 8.0×10^8^ cells ml^−1^ with yeast extract and gelatin coupled with sulfur oxidation. Other organic carbon sources did not effect on final cell yield in the presence of inorganic carbon source.

Sensitivity for antibiotics was tested under chemolithoautotrophic conditions. Growth of isolate Hiromi 1 was inhibited by the addition of erythromycin, novobiocin, tetracycline, streptomycin, chloramphenicol, vancomycin, rifampicin, kanamycin, ampicillin and penicillin G at 25 µg ml^−1^ in MMJSN medium. The strain was not sensitive to spectinomycin at 100 µg ml^−1^.

### Chemotaxonomic features

The whole-cell fatty acid analysis of strain Hiromi 1 revealed that C_16:0_ (37.1%), C_16:1_ω9c (33.1%) and C_18:1_ω11c (10.2%) were the predominant cellular fatty acids. Significant proportions of C_18:1_ω9c (7.1%), C_12:0_ (2.8%), C_14:0_ (2.4%), C_18:0_ (2.4%), C_10:0_ 3OH (1.4%), C_17:0_ (1.1%), C_12:0_ 3OH (1.1%), C_17:1_ω9 (0.6%), C_15:0_ (0.4%) and C_14:1_ω7 (0.3%) were also detected. Polar lipids of the strain Hiromi 1 include diphosphatidylglycerol (DPG), phosphatidyl ethanolamine (PE), phosphatidylglycerol (PG), ninhydrin positive phosphatidyl lipid (NPL1-2), unknown phospholipid (PL1-3), unknown glycolipid (GL) and an unknown lipid (Figure S3). The major isoprenoid quinone was Q-8.

### Phylogeny of the isolate

**Figure 2:**
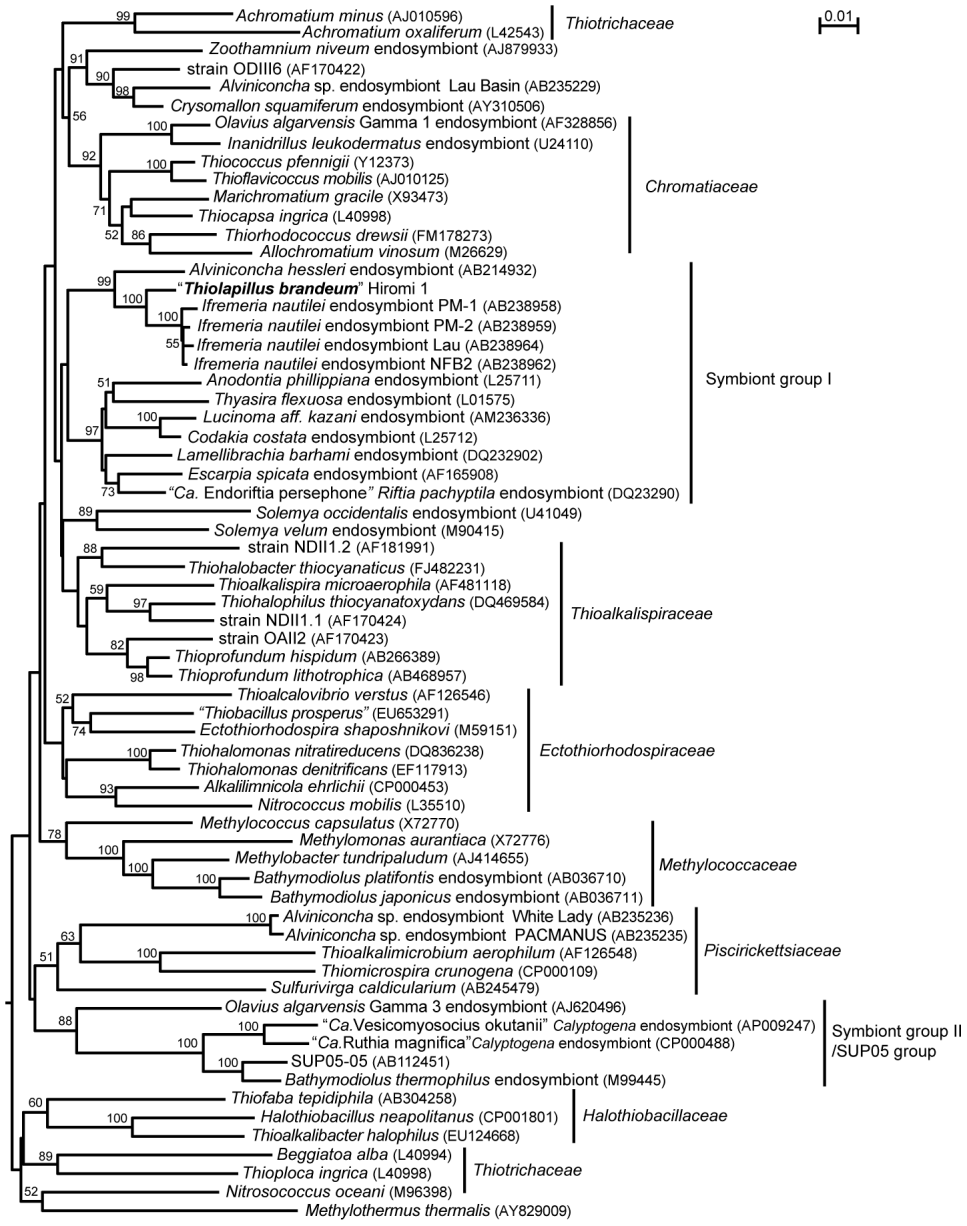
A SSU rRNA gene phylogenetic tree of chemolithoautotrophic and methanotrophic *Gammaproteobacteria* including strain Hiromi 1 constructed by the neighbor-joining method using 1253 identical positions. Bootstrap values higher than 50% are presented. GenBank/EMBL/DDBJ accession numbers are given in parentheses. Bar indicates 1 substitutions per 100 nucleotides.

The SSU rRNA gene similarity between strain Hiromi 1 and previously characterized strains is as follows: *Thioprofundum hispidum* gps61^T^ (93% similarity), *Thioprofundum lithotrophica* 106^T^ (92% similarity), *Thiorhodococcus drewsii* DSM15006 (92% similarity), *Marichromatium gracile* BN5210^T^ (92% similarity), “*Thiobacillus prosperus*” DSM5130 (91% similarity), *Thiohalomonas nitratireducens* HRHd 3sp^T^ (91% similarity), *Thiohalophilus thiocyanatoxydans* strain HRhD2^T^ (91% similarity) and *Thiohalobacter thiocyanaticus* HRh1^T^ (91% similarity). These sequence similarities of the SSU rRNA gene sequences between strain Hiromi 1 and the previously described strains fell within the common index for genus-level differentiation (90–96%) [40]. The specific close phylogenetic relationship between strain Hiromi 1 and a valid genus was not observed in the SSU rRNA gene phylogenetic analysis (Figure 2). In contrast, higher similarity values (94–97%) were obtained compared with the SSU rRNA gene sequences of deep-sea vent gastropods, such as *Ifremeria nautilei* and *Alviniconcha hessleri*, endosymbionts and an environmental SSU rRNA gene sequence obtained in a hydrothermal field in the Southern Okinawa Trough [9], [41], [42]. In the SSU rRNA gene phylogenetic tree, strain Hiromi 1 formed a robust branch with endosymbionts of *I. nautilei* and *Alviniconcha* gastropods associated with the higher hierarchy cluster of bivalve and tubeworm endosymbionts (Figure 2).

### Characterization of the isolate

Strain Hiromi 1^T^ was morphologically and physiologically distinct from the phylogenetically close *Thioalkalispiraceae* and *Chromatiaceae* species (Table S1). Long and/or spiral rods of *Thioalkalispiraceae* species and “*Thiobacillus prosperus*” were different from short straight rods of strain Hiromi 1^T^ and sphere or short rods of *Chromatiaceae* species [43]–[49]. On the other hand, cells of strain Hiromi 1^T^ harbor few intracellular large particles, while those of *Chromatiaceae* species have multiple small sulfur-like particles [45]–[48]. In *Thioalkalispiraceae*, the presence of sulfur particle was only reported in *Thioalkalispiraceae microaerophila* [50]. Phototrophy is only found in *Chromatiaceae* among the species phylogenetically close to strain Hiromi 1^T^ [45]–[48]. Chemolithoautotrophy is a common feature of *Thioalkalispiraceae* species, “*Thiobacillus prosperus*” and strain Hiromi 1^T^ [43], [44], while some of the *Chromatiaceae* species lack chemolithoautotrophic growth [45]–[49], [51], [52]. Chemoorganoheterotrophic growth was not observed in strain Hiromi 1^T^ and *Thioalkalispiraceae* species, but was observed in some of the *Chromatiaceae* species [45]–[49]. Utilization of organic compounds as carbon source was found in strain Hiromi 1^T^ and *Chromatiaceae* species under chemolithotrophic and/or phototrophic conditions, but was not observed in *Thioalkalispiraceae* species. However, the chemolithoheterotrophic growth was only tested in *Thioprofundum* species in the *Thioalkalispiraceae* [6], [44] (Table S1). Some of the *Chromatiaceae* species fix nitrogen gas, while strain Hiromi 1^T^ and *Thioalkalispiraceae* species lack the ability (Table S1). Yeast extract as a nitrogen source was only found in strain Hiromi 1^T^ and *Thioprofundum lithotrophica* [6], [44]–[54] (Table S1). Genomic G+C content of strain Hiromi 1^T^ (56.3 mol%) is relatively lower than those of *Thioalkalispiraceae* and *Chromatiaceae* species and “*Thiobacillus prosperus*” (Table S1). Optimum growth conditions, such as temperature, pH and NaCl concentration, of strain Hiromi 1^T^ were similar to *Thioalkalispiraceae* and *Chromatiaceae* species except for few alkaliphilic and acidophilic species (Table S1). Fatty acid composition of strain Hiromi 1^T^ was also similar to the both families [6], [44], [51], [53], [54] (Table S1). Based on the SSU rRNA phylogenetic analysis and distinct morphological and physiological features of strain Hiromi 1^T^, we propose a novel genus and species “*Thiolapillus brandeum*” represented by the type strain Hiromi 1^T^ ( = JCM15507^T^, DSM23672^T^).

### General genomic features

The genome of strain Hiromi 1 consists of a single circular chromosome (3,129,661 bp) and two plasmids (pTBH1 and pTBH2; 10,872 and 13,616 bp, respectively). Totals of 2,922, 8 and 18 CDSs were identified in the chromosome and the two plasmids, with 56.3, 54.2 and 50.1% G+C content, respectively. Among CDSs in the chromosome and two plasmids, 1,840, 4 and 7 CDSs could be assigned to certain functions, 643, 0 and 7 CDSs could be identified as hypothetically conserved proteins, and the remaining 438, 4 and 4 CDSs did not show significant similarity to any amino acid sequence in protein databases, respectively. One rRNA gene operon and 38 tRNA genes were identified. The genome contains one region of clustered regulatory interspaced short palindromic repeats (CRISPR) with 2,291 bp, and a CRISPR-related gene cluster followed the repeats.

### Central metabolism

**Figure 3:**
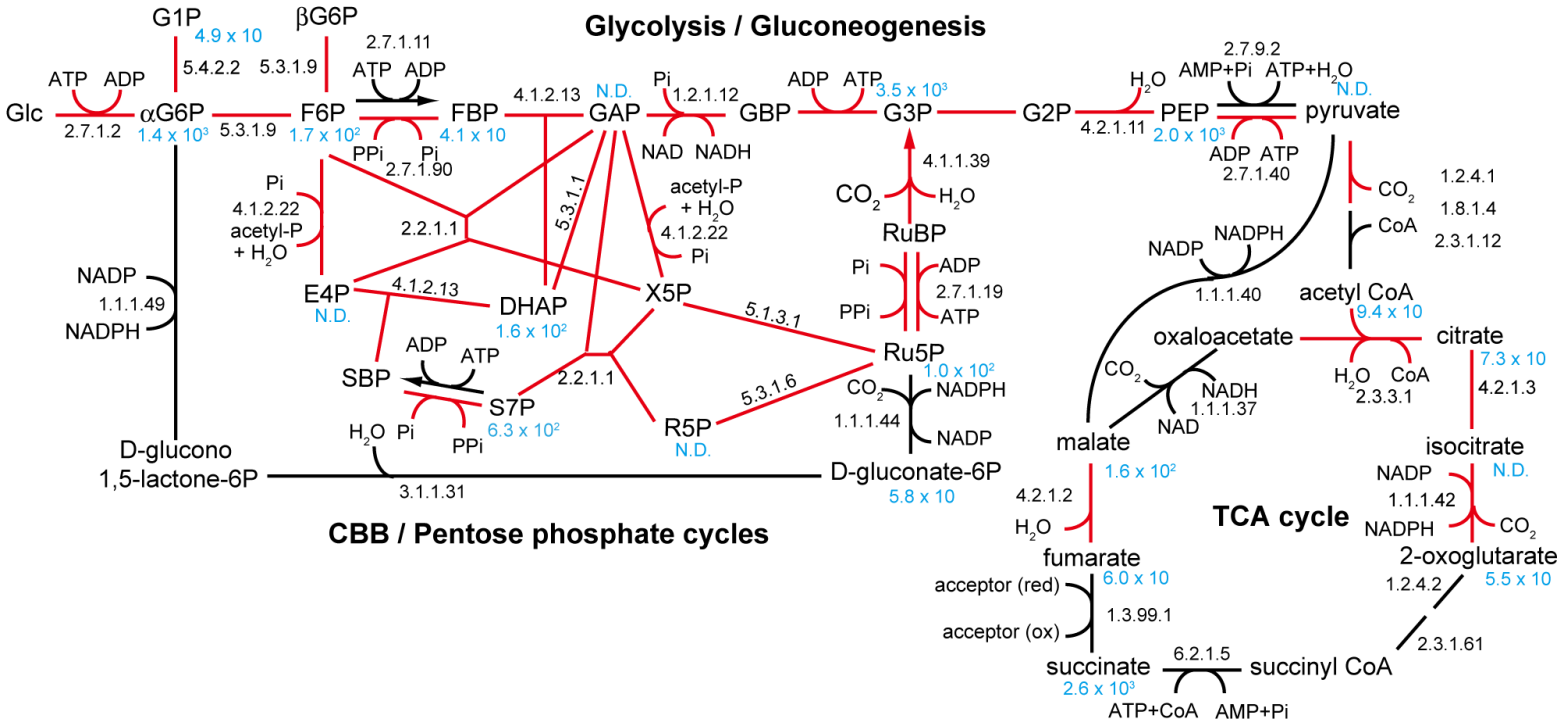
Predicted central metabolism of strain Hiromi 1. Red lines indicate conserved pathways in most of the publically accessible genomes of the chemolithoautotrophic *Gammaproteobacteria*. EC numbers are given on each enzymatic reaction. Light blue font indicates concentrations (per pmol 10^10^ cells) of metabolites. Metabolites that were not targets of the metabolomic analysis were given neither concentrations nor N.D. N.D.; not detected. Pi, phosphate; PPi, pyrophosphate; Glc, α-D-glucose; αG6P, α-D-glucose-6-phosphate; G1P, α-D-glucose-1-phosphate; βG6P, β-D-glucose-1-phosphate; F6P, β-D-fructose-6-phophate; FBP, β-D-fructose-1,6-bisphosphate; GAP, glyceraldehyde-3-phosphate; GBP, glycerate-1,3-bisphosphate; G3P, glycerate-3-phosphate; G2P, glycerate-2-phosphate; PEP, phosphoenolpyruvate; E4P, erythrose-4-phosphate; acetyl-P, acetyl phosphate; DHAP, dihydroxyacetone phosphate; X5P, D-xylulose-5-phosphate; SBP, D-sedoheptulose-1,7-bisphosphate; S7P, D-sedoheptulose-7-phosphate; RuBP, ribulose-1,5-bisphosphate; Ru5P, ribulose-5-phophate; R5P, D-ribose-5-phosphate.

The genes for nearly complete Embden-Meyerhof-Parnas pathway, and Calvin-Benson-Bassham (CBB) and pentose phosphate cycles are present, but genes for the Entner-Doudroff pathway are absent in the genome of this strain (Figure 3). The strain has one form II ribulose-1,5-bisphosphate carboxylase/oxygenase (Rubisco), and its enzymatic activity was estimated to be 0.01 µmol min^−1^/mg protein in the cell-free extract at 37°C. One carbonic anhydrase is encoded in the genome. The genome harbors pyrophosphate-dependent 6-phosphofructokinase (PPi-PFK) and proton-translocating pyrophosphatase, which have been identified in methanotrophic and thiotrophic *Gammaproteobacteria* [35], [55], [56]. However, typical bacterial fructose 1,6-bisphosphatases (class 1 and 2) and their possible alternatives, such as archaeal ADP-dependent phosphofructokinase [57] and recently identified fructose 1,6 bisphosphate aldolase/phosphatase [58], were not identified. We checked the enzymatic activities of bacterial 6-phosphofructokinases and fructose 1,6-bisphosphatases in the cell-free extract prepared from cells grown chemolithoautotrophically. Consumption and production activities of fructose 6-phoshate by PPi-PFK were determined to be 0.66 and 0.15 µmol/min/mg protein, respectively, in the cell-free extract at 37°C. Interestingly, ATP-dependent 6-phosphofructokinase activity was not detected, although the gene is encoded in the genome. PPi-PFK is also known to function as an alternate of a dual functional enzyme, class 2 fructose 1,6-bisphosphatase [35]. PPi-PFK is conserved in all complete genomes of sulfur-oxidizing *Gammaproteobacteria* in public databases, while all of these genomes lack the bifunctional fructose 1,6-bisphosphatase class 2/sedoheptulose 1,7-bisphosphatase genes that have been believed to be essential in the CBB cycle [59]. Accordingly, PPi-PFK could be the key functional enzyme of the central carbon metabolism in these organisms. The strain does not harbor transaldolase gene(s), although transaldolase is one of the representative enzymes in the pentose phosphate pathway. The absence of transaldolase is also observed in the genomes of *T. crunogena*, *Calyptogena* symbionts, *Beggiatoa* sp. and “*Ca.* E. persephone”. Fructose-bisphosphate aldolase/sedoheptulose-1,7-bisphosphate aldolase and PPi-PFK/sedoheptulose-7-phosphatekinase most likely substitute for the function of the transaldolase.

Genes for complete tricarboxylic acid (TCA) cycle are present. Co-occurrence of the reductive TCA and CBB cycles has only been found in endosymbionts of tubeworms inhabiting hydrothermal environments [60], [61], but this was not observed in this strain. The TCA cycle of this strain does not have a glyoxylate bypass. Genes for malate dehydrogenase and pyruvate carboxylase, which may contribute to anaplerotic carbon fixation, are present, but a gene for phosphoenolpyruvate carboxylase is absent. Genes for 3-hydroxypropionate pathway found in a sulfur-oxidizing gammaproteobacterial symbiont in gutless worms were not identified [55], [56]. Despite a nearly complete set of glycolysis pathway and TCA cycle genes, the strain does not grow with sugars as the sole carbon source. Organic acids such as succinate, fumarate and pyruvate and amino acids digested from peptides are likely assimilated through the TCA cycle.

The assimilation of organic acids may have advantages in the synthesis of cellular NADPH, although NAD(P)H may also be produced from the processes associated with sulfur oxidation pathway. The pentose phosphate pathway (oxidative branch) is known to be the major pathway for NADPH production and well conserved in the genomes of sulfur-oxidizing *Gammaproteobacteria*, but the genomes of several, such as *Calyptogena* endosymbionts, *Alkalilimnicola ehrlichei*, *Thioalkalivibrio* strain K90mix and *Halorhodospira halophila*, do not possess the branch. In contrast, the portion of the TCA cycle contributing NADPH production is conserved in all the genomic sequences in chemolithoautotrophic *Gammaproteobacteria* that grow with carbon fixation through the CBB pathway. The distribution pattern of both pathways in chemolithoautotrophic *Gammaproteobacteria* suggests that the TCA cycle would play significant roles in NADPH production as compared with the pentose phosphate pathway (oxidative branch). Accordingly, organic acid assimilation through the TCA cycle is advantageous not only for saving energy for carbon fixation but also for the production of NADPH.

### Energy metabolism

**Figure 4:**
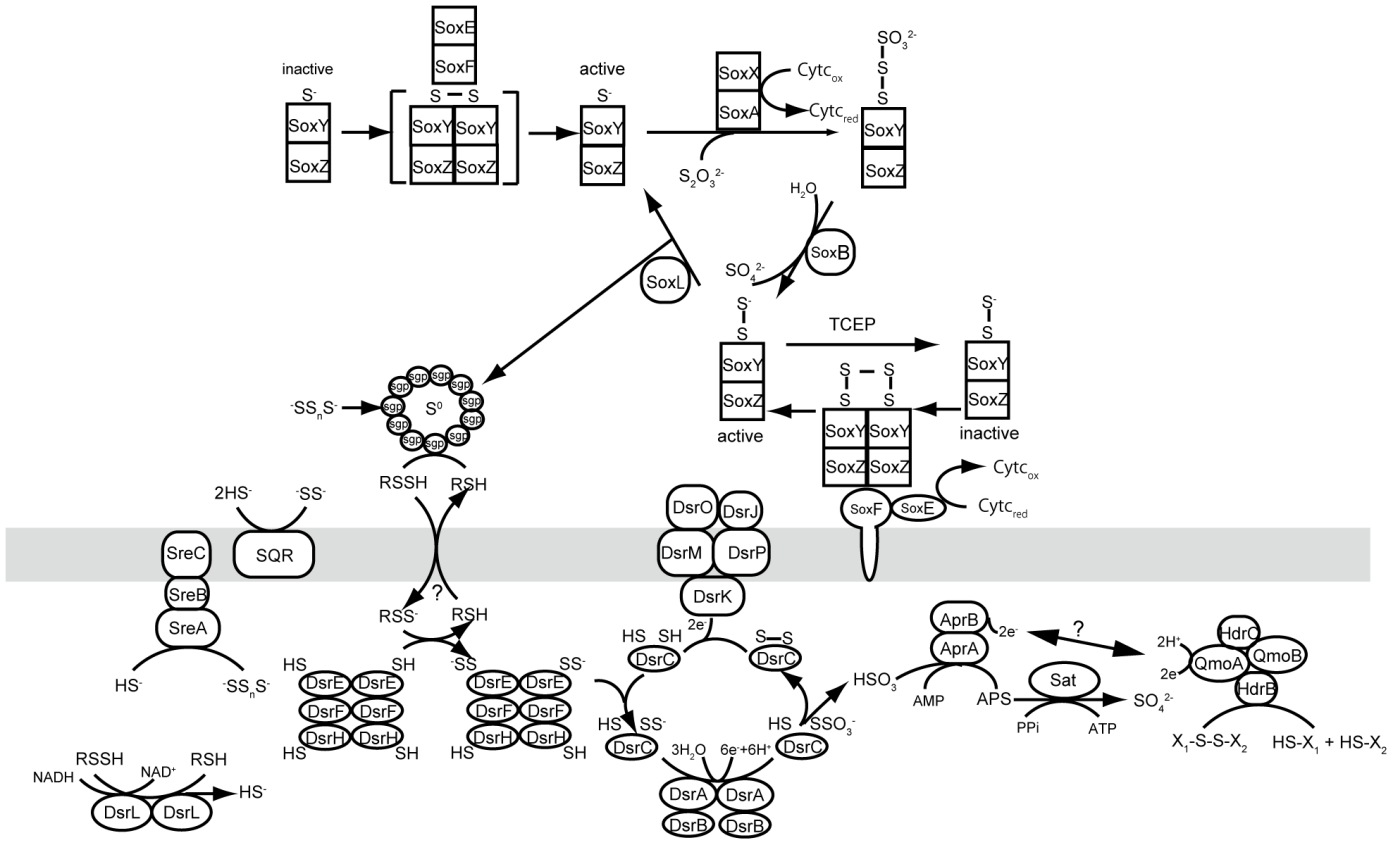
Predicted sulfur oxidation pathways of strain Hiromi 1 inferred from the previously published literatures [63], [66]–[68], [74], [75]. TCEP; tris(2-carboxyethyl)phosphine.

Genes that are necessary for the physiological features of the novel isolate, sulfur-oxidation coupled with aerobic respiration and denitrification, were identified in the genome. Genes for an incomplete-type Sox system and a nearly complete Dsr system for oxidation of sulfur compounds such as sulfide, elemental sulfur, thiosulfate and sulfite, are present in the genome, as is the case for several sulfur-oxidizing *Gammaproteobacteria* (Figure 4). Among the genes for periplasmic Sox-type sulfur oxidation system, genes for SoxBZYKAX are encoded in a gene cluster, but the genes for SoxCD and their homologues SorAB are missing, as is the case of the incomplete SOX system in other sulfur-oxidizing *Gammaproteobacteria*. A gene for SoxL most likely functions in the turnover of SoxYZ complex [62], and three genes for SoxW were identified in the genome. SoxCD and their respective homologues SorAB are missing, as are SoxVGJ. One operon-like gene cluster for flavoprotein-cytochrome *c* complex (SoxEF), which catalyzes sulfide oxidation and activation of SoxYZ complex, is encoded in the genome [63]. In addition to the Sox proteins, other periplasmic proteins related to sulfur oxidation, such as three homologues of sulfur globule proteins (Sgp) [64], and at least one sulfide:quinone oxidoreductase were identified in the genome (Figure 4).

A complete set of Dsr system genes, which are responsible for cytoplasmic sulfur oxidation, is encoded in the same order as found in *Alc. vinosum* (*dsrABEFHCMKLJOPNRS*) [65]–[67] (Figure 4). Genes for catalytic subunits of adenylsulfate reductase AprAB are present, but the membrane-binding subunit AprM or its alternative membrane binding QmoABC complex [66], [68]–[72], which is responsible for electron transfer, is absent. However, interestingly, the genome possesses an operon-like gene cluster of *qmoAB-hdrBC* that is not adjacent to the *aprAB* operon-like gene cluster. HdrBC are the subunits of heterodisulfide reductase, QmoAB are homologues of HdrA, and HdrC is the homologue of the N-terminal non-transmembrane region of QmoC [68]. Thus, a putative QmoAB-HdrBC complex is most likely the chimera of QmoABC and heterodisulfide reductase; such gene organization is also be observed in some genomes of *Chlorobi* species and *Betaproteobacteria Thiobacillus denitrificans*, although the *in situ* function of the complex is not clear. A gene for ATP sulfurylase (Sat) is encoded in the genome. Moreover, the strain harbors genes for a homologue of sulfur reductase (SreABC) that has been identified in *Aquifex aeolicus* [73], [74], although the strain lacks the gene for a hydrogenase that forms a super-complex with SreABC in *A. aeolicus* (Figure 4).

Gammaproteobacterial sulfur-oxidizing pathways have been intensively studied biochemically in the anaerobic phototroph *Allochromatium* [64], [75], [76]. In contrast, knowledge about those pathways in facultatively or obligately aerobic chemolithotrophic sulfur oxidizers in *Gammaproteobacteria* is limited to sequence-based approaches [60], [77]–[81]. Based on these analyses it has been recognized that most of the chemotrophic sulfur-oxidizing *Gammaproteobacteria* harbor both incomplete Sox and complete Dsr systems, similar to the phototrophic *Chromatiaceae* gammaproteobacterial species [3] with one exception; *Thiomicrospira crunogena* possess a complete Sox system but lacks the Dsr system [77]. Strain Hiromi 1 harbors incomplete Sox and complete Dsr systems, as do the typical gammaproteobacterial sulfur-oxidizers. Genes for the flavoprotein-cytochrome *c* complex (SoxEF) are present in strain Hiromi 1 and *Chromatiaceae* species but are absent in *Calyptogena* clam symbionts and *T. crunogena*.

The strain harbors the gene components for complete aerobic respiratory chain consisting of complex I, II and III and cytochrome *cbb_3_*-type cytochrome *c* oxidase. A complete denitrification pathway including two membrane-bound periplasmic nitrate reductase (Nap), two cytochrome *cd*-type nitrite reductases (NirS), a set of membrane-bound nitric oxide reductase (Nor), and a set of nitrous oxide reductase (NosZ) was also identified in the genome (Figure S4).

In addition, genes for formate hydrogenlyase, a complex homologous to *E. coli* hydrogenase-4, are found in the genome. The putative formate hydrogenlyase lacks a ferredoxin subunit (HyfA), an electron accepting/donating component in oxidoreduction pathways [82], suggesting the putative enzyme forms a supercomplex with other oxidoreductases that have a ferredoxin subunit as an electron acceptor/donator. The absence of H_2_ uptake hydrogenases that were identified from some sulfur oxidizers associated with hydrothermal ecosystem [60], [77], [83], [84] correlated with its incapability of hydrogenotrophic growth. Ferredoxin:NAD^+^ oxidoreductase Rnf and F-type ATPase genes are present.

### Nitrogen metabolism and amino acid synthesis

Genes for nitrogenase and assimilatory nitrate and nitrite reductases are absent, which is consistent with the inability of strain Hiromi 1 to use nitrate and N_2_ as inorganic nitrogen sources, as shown by the cultivation experiments. Biosynthetic pathways for all 20 amino acids necessary for protein synthesis were identified in the genome (Figure S5). The amino acid biosynthetic pathways and glutamine synthetase are present, but genes for another representative ammonia-assimilating enzyme, glutamate dehydrogenase, are missing. Thus, alanine dehydrogenase, serine deaminase, threonine dehydrogenase and cystein synthetase could be responsible for ammonia assimilation in amino acid biosynthesis. In addition, genes for phosphoserine phosphatase are missing in the genome. Because metabolomic analysis of the chemolithoautotrophically grown cells revealed that glutamate was present as the most abundant cellar amino acid component among the amino acids (Figure S5), alanine dehydrogenase is most likely the key enzyme for ammonium assimilation in this organism. Genes for urease are absent.

### Motility, sensor, chemotaxis and signaling

Although neither motility nor flagella have been observed in microscopic observations of this strain, an almost complete gene set for flagellar biosynthesis and flagellar proteins is found in one genomic region (Text S1). Nearly complete genes for type IV pilus formation are scattered throughout the genome. The strain possesses two chemotaxis-like gene clusters. One gene cluster encodes chemotaxis system proteins related to motility, such as CheAWRBY and methyl-accepting chemotaxis protein (MCP) [85], [86]. The other is a homologous gene cluster of the Wsp chemosensory system. The Wsp system in *Pseudomonas aeruginosa* regulates biofilm formation [87], and the presence of the system is consistent with the biofilm formation of this strain under static cultivation condition.

Other than the genetic components of flagella formation, chemotaxis and pilus formation, the genome contains more than 20 two-component signal transduction systems (TCSs) composed of histidine kinases and their response regulator substrates. Intriguingly, genes are present for two TCSs homologous to QseB/QseC and one to QseE/QseF, which are parts of quorum sensing systems linking autoinducer and host hormone sensing in virulent *Gammaproteobacteria* [88]–[90]. In addition, genes for the TCS of RpfC/RpfG, part of a diffusible signaling factor (DSF) signaling network that consists of RpfF, RpfC, RpfG and Clp, were also identified in the genome. The TCS of RpfC/RpfG found in *Xanthomonas campestris* modulates functions related to virulence and adaptation [91]. Other TCS genes potentially involved in other quorum sensing systems, such as *lasRI* and *rhlRI* in *Pseudomonas aeruginosa* [92] and *cqsS-luxU* and *luxQU* in *Vibrio cholerae* [93], are absent. In addition, TCSs homologous to PhoB/PhoR, PhoP/PhoQ, BarA/UvrY, EnvZ/OmpR, RstA/RstB, CbrA/CbrB, AlgZ/AlgR and NarL/NarX that may relate to virulence traits were identified while capability of virulence has not been observed in this strain (Text S1).

### Transporters and secretion system

As potential adaptation strategy for metal-rich deep-sea hydrothermal environments, it is predicted that the strain has multiple efflux systems and transporters for heavy metals, some of which would be horizontally derived from other microbial components living in similar habitats. Potential horizontally acquired systems do not show significant sequence similarity with the gammaproteobacterial entities but are related to the homologues from other proteobacterial classes, *Firmicutes* and/or deeply branching *Bacteria* and *Archaea* that have been observed in hydrothermal environments (Text S1). The genome harbors genes for amino acid transporters, a glutamate symporter and an oligopeptide transporter, although genes for sugar transporters were not found. The presence and absence of genes for these transporters is consistent with the substrate utilization of the new isolate, such as the capability to grow with various organic acids and proteinaceous compounds but not with sugars as the carbon source.

Gene sets of type I and II secretion systems, lipoprotein-releasing ABC transporter LolCD complex and periplasmic chaperon LolA were found (Text S1). Genes for a lipopolysaccharide export system and outer membrane phospholipid importing system are present. In addition, genes for biopolymer transport system including the protein Pal (peptidoglycan-associated lipoprotein), which is considered essential for bacterial survival and pathogenesis, were also identified. The biopolymer transport system is most likely involved in biogenesis and/or transport of lipopolysaccharide components, in the formation of cell envelopes in daughter cells and in the uptake/transport of compounds through the cytoplasmic membrane [94] (Text S1).

### Ecological functions of a symbiotic lineage of sulfur-oxidizing *Gammaproteobacteria*

Physiology and genomic features of strain Hiromi 1 provide novel insights into the function and niche adaptation of sulfur-oxidizing *Gammaproteobacteria* that belongs to the previously uncultivated symbiont-related lineage in hydrothermal ecosystems. Sulfur-oxidizing *Gammaproteobacteria* have been known as one of the major primary producers in hydrothermal environments especially mixing zones of hydrothermal fluids and deep-sea water, in which chemosynthetic animals such as polychaetes, mussels, gastropods and shrimps distribute [1]–[3]. To date, the chemolithoheterotrophic growth of sulfur-oxidizing chemolithoautotrophic lineages of *Gammaproteobacteria* in hydrothermal environments was only reported in *Thiomicrospira* phylogenetically distinct from endosymbionts [77], [95] while mixotrophy in the symbiotic lineages was suggested by metagenomic analyses of the *Riftia* tubeworm endosymbiont “*Ca*. E. persephone” and scaly-foot gastropod endosymbionts [84], [96]. Many genes for chemotaxis, and virulence-and quorum sensing-related sensors in the genome of Hiromi 1 are consistent with its mixotrophic growth characteristics, although flagella formation was not observed under any of the cultivation conditions in this study. The genetic repertoire suggests that strain Hiromi 1 may induce flagella formation when the sensing systems discern specific ecophysiological states, such as depletion of specific energy and carbon sources and expression of bacterial autoinducers and hormone from the adjacent animals. Utilization of organic carbons and nitrogens in strain Hiromi 1 imply that sensing and chemotaxis for the hydrothermal vent animals and microbial biofilm formation would serve to access the organic substrates for additional carbon sources that enhance ATP saving for carbon fixation and NADPH production. Such positive behavior of free-living microbes towards animal body may significantly increase the physical contact between microbes and animal body. These contacts could be important triggers to establish the chemosynthetic symbioses in deep-sea hydrothermal environments considering the close phylogenetic relationship between *T. brandenum* and gastropods endosymbionts. Moreover, the cultivation and genomic analysis in this study support the hypothesis that mixotrophy of endosymbionts and bidirectional nutrition interaction between endosymbionts and host animal occur based on metagenomic analysis.

The polysaccharide-like substance secretion of the chemolithoautotrophic sulfur-oxidizer would also provide novel insights into nutrient cycle in hydrothermal ecosystem. Organic carbon secretion may contribute in extracellular organic carbon pool of adhesive microbial ecosystems in hydrothermal environments. Heterotrophic organisms, hyperthermophilic archaea to mesophilic bacteria, also dominate in hydrothermal vent ecosystem as well as chemolithoautotrophic organisms [1], but the organic carbon supply from primary producers is poorly understood in hydrothermal ecosystems. In general, cell lysis by viral infection plays a major role in marine nutrient cycles [97]. However, relatively low viral abundance toward adhesive microbial populations was observed in hydrothermal environments [98]. The observation presents the possibility that biofilm secretion from chemolithoautotrophs in hydrothermal environments may play more important roles in nutrient cycle compared to other marine ecosystems. In addition, the biofilm secretion in this strain suggests that the organic compound secretion of symbionts could be a supplemental nutritional interaction from the symbionts to the host animals in addition to on demand digestion of endosymbionts.

### Description of *Thiolapillus* gen. nov

Etymology: Thi.o.la.pil.lus. Gr. n. *thios* sulfur; L. n. *lapillus* precious stone; N.L. neut. Nn. (sulfur oxidizer with precious stone referring to the cytoplasmic globule structure);

Short rods. Facultatively anaerobic and neutrophilic. Chemolithoautotrophic and chemolithoheterotrophic. Able to utilize reduced sulfur compounds as electron donors and nitrate and molecular oxygen as electron acceptors. NaCl is required for growth. Based on the SSU rRNA gene sequence, *Ifremeria nautilei* endosmbionts are likely characterized in the genus *Thiolapillus.*

### Description of *Thiolapillus brandeum* gen. nov. sp. nov

Etymology: bra.n.de'um. L. n. *brandeum* shroud referring to biofilm formation covering elemental sulfur).

Non-motile short rods, 0.45–1.2×1.3–4.0 µm. Facultatively anaerobic. Growth occurs at 25–45°C (optimum 40°C), pH 5.7–8.4 (optimum 6.5) and 2.0–4.5% (optimum 3.0%) NaCl. Sulfur, thiosulfate and tetrathionate are utilized as electron donors, and oxygen and nitrate are utilized as electron acceptors. Sulfate is produced. Ammonium and yeast extract are utilized as nitrogen source. Fumarate, formate, citrate, pyruvate, succinate and yeast extract, peptone, tryptone peptone, casamino acids and gelatin are utilized as carbon sources coupled with sulfur oxidation. Sensitive to erythromycin, novobiocin, tetracycline, streptomycin, chloramphenicol, vancomycin, rifampicin, kanamycin, ampicillin and penicillin G but not sensitive to spectinomycin. The major polar lipids are diphosphatidylglycerol, phosphatidyl ethanolamine, phosphatidylglycerol, ninhydrin positive phosphatidyl lipid and unknown phospholipid. The major isoprenoid quinone is Q-8. The dominant cellular fatty acids are C_16:0_, C_16:1_ω9c, C_18:1_ω11c and C_18:1_ω9c. Type strain is Hiromi 1^T^ ( = JCM15507^T^, DSM23672^T^), isolated from the chimney structure at the Minami-Ensei Knoll hydrothermal field, Okinawa Trough. The genomic (chromosomal) G+C content of the type strain is 56.3 mol%.

## Supporting Information

Figure S1 **A scanning micrograph of broken polysaccharide-like substance layer on elemental sulfur.** Bar, 1 µm.
(TIFF)

Figure S2 **Effects of temperature, pH, NaCl and O_2_ on the growth of strain Hiromi 1.**
(TIF)

Figure S3 **Polar lipids profile of strain Hiromi 1 after separation by two-dimensional TLC.** DPG, diphosphatidylglycerol; PE, phosphatidyl ethanolamine; PG, phosphatidylglycerol; NPL1-2, ninhydrin positive phosphatidyl lipid; PL1-3, unknown phospholipid; GL, unknown glycolipid; L, unknown lipid.
(TIF)

Figure S4 **Predicted respiratory chains in strain Hiromi 1.**
(TIF)

Figure S5 **Predicted synthetic pathways for 20 amino acids.** Amino acids are shown in red or orange font. EC numbers are given on each enzymatic reaction. Blue fonts indicate the concentrations of the metabolites (per pmol 10^10^ cells). N.D., not detected; P, phosphate.
(TIF)

Table S1 **Characteristics of strain Hiromi 1 and its relatives based on the SSU rRNA gene sequence similarity.**
(PDF)

Text S1 **Additional details on the genomic information of the strain.**
(DOC)
