# Supplementary figures and images for: Physiological and Genomic Features of a Novel Sulfur-Oxidizing Gammaproteobacterium Belonging to a Previously Uncultivated Symbiotic Lineage Isolated from a Hydrothermal Vent
Source: PLoS One. 2014 Aug 18;9(8):e104959. doi: 10.1371/journal.pone.0104959 (PMC4136832; doi:10.1371/journal.pone.0104959)

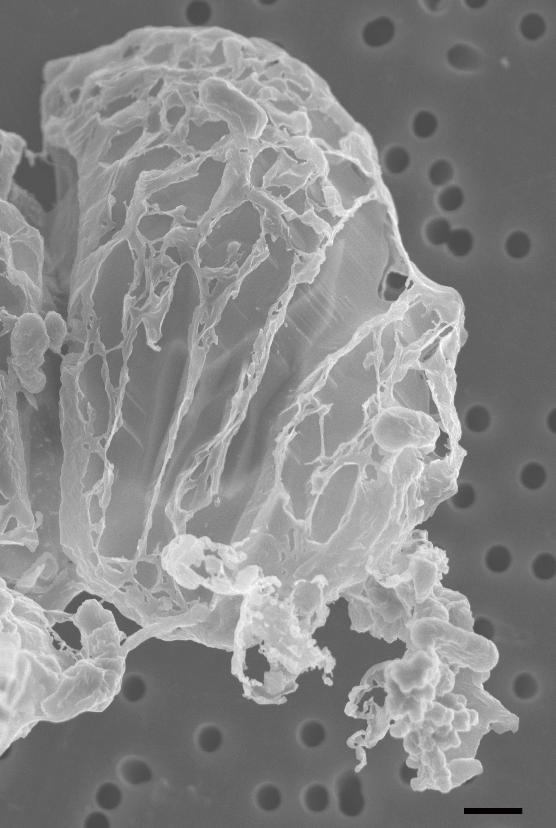

Supplement: Figure S1 — A scanning micrograph of broken polysaccharide-like substance layer on elemental sulfur. Bar, 1 µm. (TIFF) [file pone.0104959.s001.tiff]

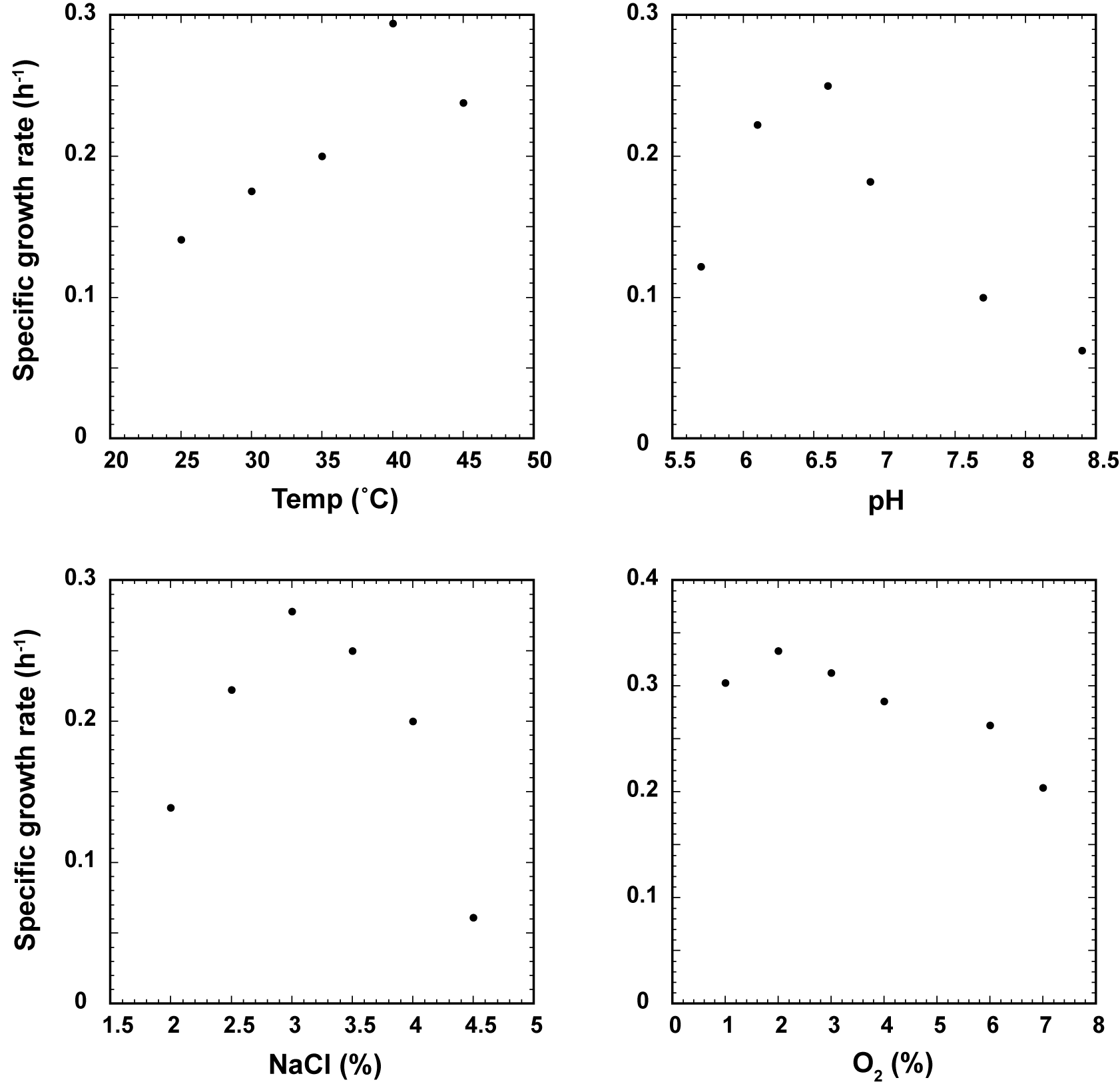

Supplement: Figure S2 — Effects of temperature, pH, NaCl and O2 on the growth of strain Hiromi 1. (TIF) [file pone.0104959.s002.tif]

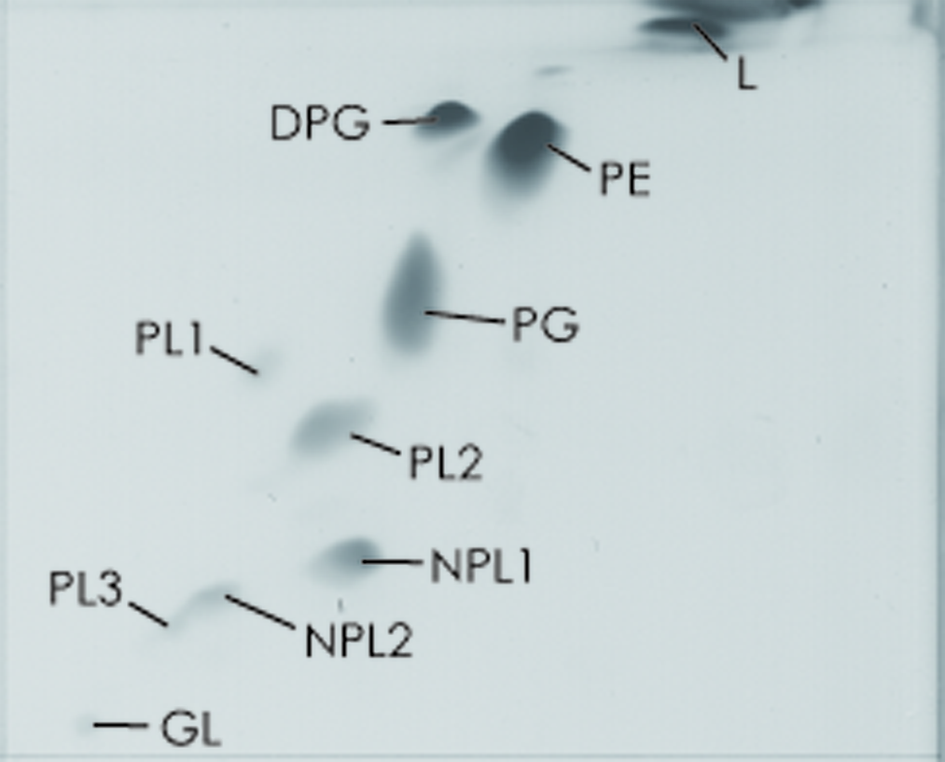

Supplement: Figure S3 — Polar lipids profile of strain Hiromi 1 after separation by two-dimensional TLC. DPG, diphosphatidylglycerol; PE, phosphatidyl ethanolamine; PG, phosphatidylglycerol; NPL1-2, ninhydrin positive phosphatidyl lipid; PL1-3, unknown phospholipid; GL, unknown glycolipid; L, unknown lipid. (TIF) [file pone.0104959.s003.tif]

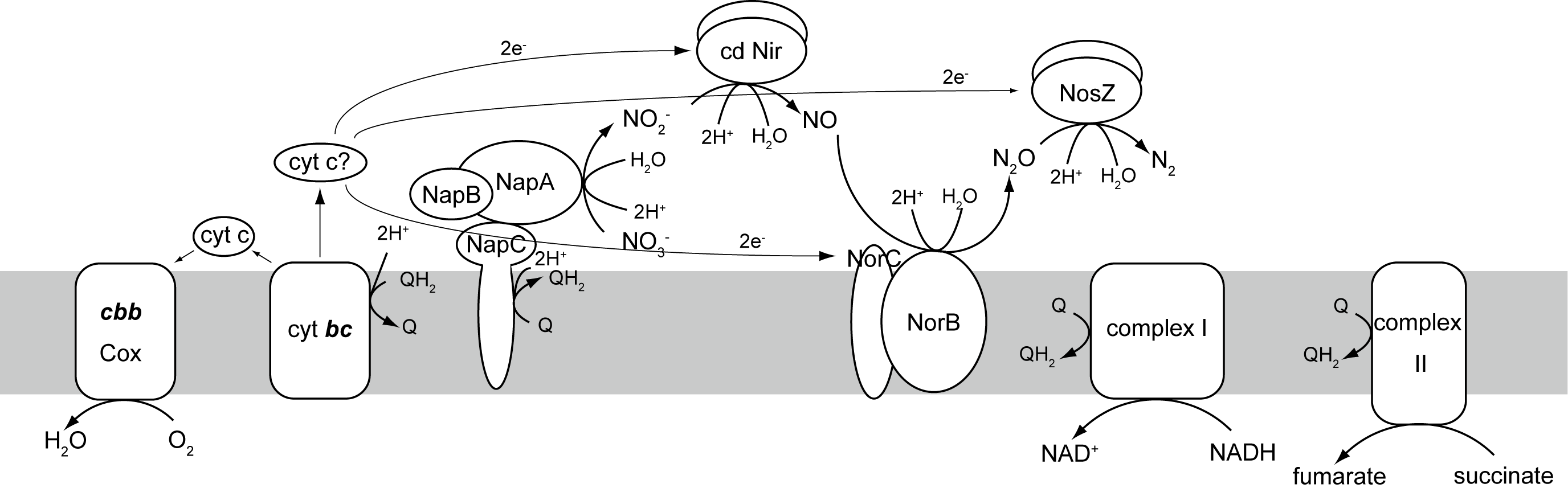

Supplement: Figure S4 — Predicted respiratory chains in strain Hiromi 1. (TIF) [file pone.0104959.s004.tif]

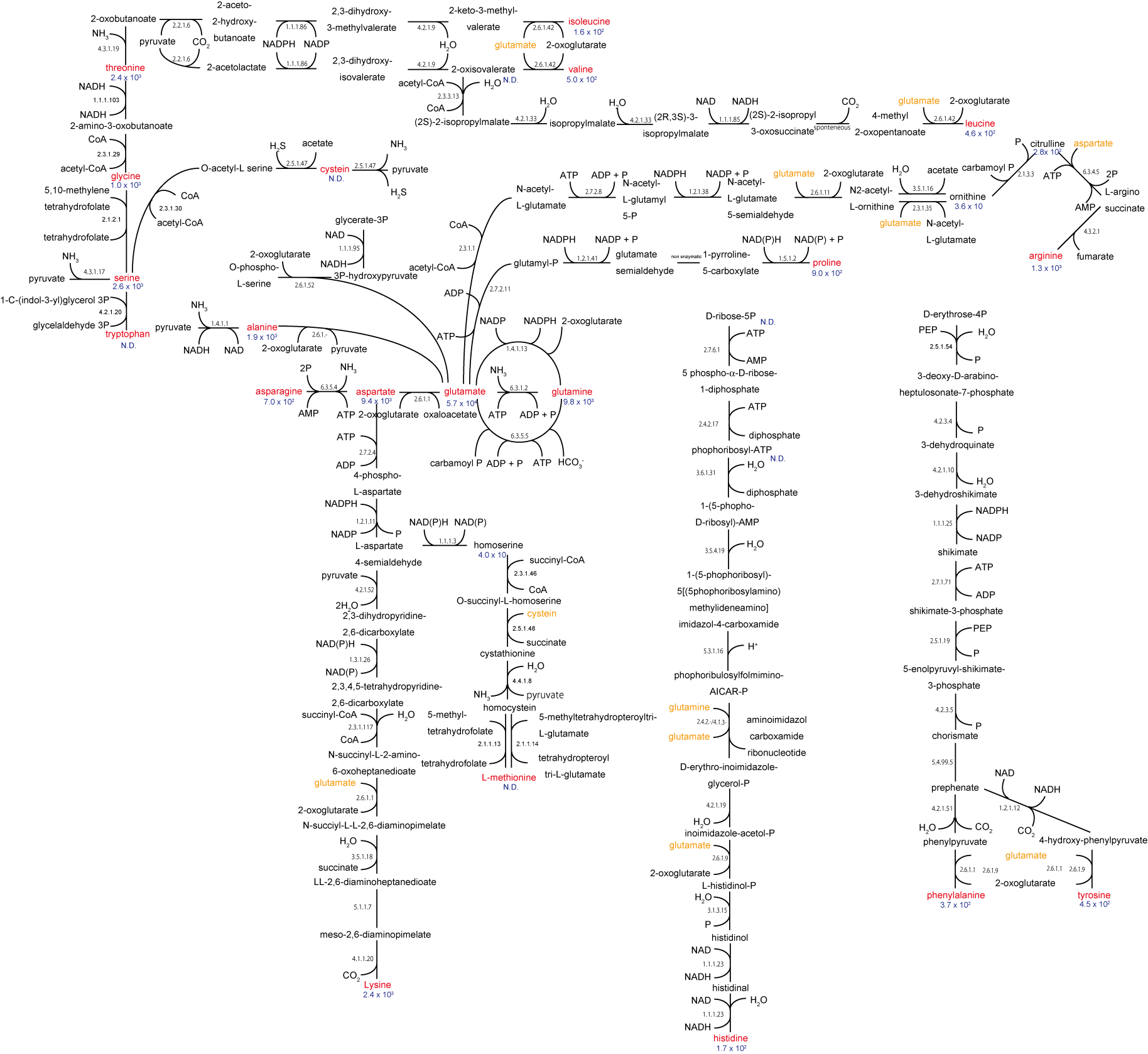

Supplement: Figure S5 — Predicted synthetic pathways for 20 amino acids. Amino acids are shown in red or orange font. EC numbers are given on each enzymatic reaction. Blue fonts indicate the concentrations of the metabolites (per pmol 1010 cells). N.D., not detected; P, phosphate. (TIF) [file pone.0104959.s005.tif]
