# Supplementary material for: Physiological and Genomic Features of a Novel Sulfur-Oxidizing Gammaproteobacterium Belonging to a Previously Uncultivated Symbiotic Lineage Isolated from a Hydrothermal Vent
Source: PLoS One. 2014 Aug 18;9(8):e104959. doi: 10.1371/journal.pone.0104959 (PMC4136832; doi:10.1371/journal.pone.0104959)
Supplement: Table S1 — Characteristics of strain Hiromi 1 and its relatives based on the SSU rRNA gene sequence similarity. (PDF) [file pone.0104959.s006.pdf]

Table S1. Characteristics of strain Hiromi 1 and its relatives based on SSU rRNA gene sequence similarity.

	family incertae sedis	*Thioalkalispiraceae*				
Genus	*Thiolapillus*	*Thioprofundum*	*Thioprofundum*	*Thiohalobacter*	*Thioalkalispira*	*Thiohalophilus*
Species	*brandeum*	*lithotrophica*	*hispidum*	*thiocyanaticus*	*microaerophila*	*thiocyanatoxydans*
Strain	Hiromi1	106^T	gps61^T	HRh1^T		
Cell shape	rod	long spiral rod	rod	rod	spiral rod	rod
Cell size (μm)	0.45–1.2 x 1.3–4.0	0.4-0.6 x 1-20	0.3 x 1.5-2.0	0.4 x 1.0-2.0	0.3-0.45 x 1-4	0.35-0.4 x 2-6
Motility	-	+	+	-	+	-
Intracellular globules	+ (1-2)	-	-	N.D.	+	-
G+C content (mol %)	56.3 (genome, chromosome)	66 (HPLC)	62.9 (HPLC)	63.5 (Tm)	58.9 (Tm)	58.2 (Tm)
Temperature for growth (˚C)						
range	25-45	30-55	29-43	20-40	N.R.	N.R.
optimum	40	50	39	32	(30)	(30)
pH for growth						
range	5.7-8.4	5.8-7.6	6.0-8.0	6.3-8.0	8.0-10.4	6.5-8.2
optimum	6.5	7.0	7.0	7.3-7.5	10	7.5
NaCl concentration for growth (%)						
range	2.0-4.5	1.2-4.2	1.0-4.0	1.2-17.5	1.2-8.2	0.6-23.4
optimum	3.0	3.0	2.0	2.9	2.9	8.8
Chemolithoautotrophy	+	+	+	+	+	+
Electron donors for chemolithotrophy	S^0, $S_2O_3^{2-}$, $S_4O_6^{2-}$	S^0, $S_2O_3^{2-}$, SO_3^-, $S_4O_6^{2-}$	S^0, $S_2O_3^{2-}$, $S_4O_6^{2-}$	S^0, HS^-, $S_2O_3^{2-}$, $S_4O_6^{2-}$, SCN^-	S^0, HS^-, $S_2O_3^{2-}$	HS^-, $S_2O_3^{2-}$, SCN^-
Electron acceptors for chemotrophy	O_2, NO_3^-	O_2, NO_3^-	O_2, NO_3^-	O_2	O_2	O_2, NO_2^-
Phototrophy	-	-	-	-	-	-
Inorganic electron donors for phototrophy						
Mixotrophy	+	-	N.D.	N.D.	N.D.	N.D.
Chemoorganoheterotrophy	-	-	-	-	-	-
Organic carbon sources /organic electron donors			*			
Formate	+	-	-	N.D.	N.D.	N.D.
Acetate	-	-	-	N.D.	N.D.	N.D.
Pyruvate	+	-	-	N.D.	N.D.	N.D.
Propionate	-	N.D.	N.D.	N.D.	N.D.	N.D.
Butyrate	N.D.	N.D.	-	N.D.	N.D.	N.D.
Lactate	-	N.D.	-	N.D.	N.D.	N.D.
Fumarate	+	N.D.	-	N.D.	N.D.	N.D.
Succinate	+	N.D.	-	N.D.	N.D.	N.D.
Citrate	+	N.D.	-	N.D.	N.D.	N.D.
Malate	-	N.D.	-	N.D.	N.D.	N.D.
Fructose	-	N.D.	-	N.D.	N.D.	N.D.
Glucose	-	N.D.	-	N.D.	N.D.	N.D.
Ethanol	-	N.D.	N.D.	N.D.	N.D.	N.D.
Propanol	N.D.	N.D.	N.D.	N.D.	N.D.	N.D.
Glycerol	-	N.D.	N.D.	N.D.	N.D.	N.D.
Yeast Extract	+	-	N.D.	N.D.	N.D.	N.D.
Tryptone peptone	+	-	N.D.	N.D.	N.D.	N.D.
Peptone	+	N.D.	N.D.	N.D.	N.D.	N.D.
Casamino Acids	+	-	N.D.	N.D.	N.D.	N.D.
Gelatin	+	N.D.	N.D.	N.D.	N.D.	N.D.
Nitrogen sources	NH_4^{+}†, yeast extract	N_2, NH_4^+, NO_3^-, yeast extract	NH_4^{+}#	NH_4^{+}#	NH_4^+, NO_3^-, NO_2^-	NH_4^{+}#
Major fatty acids	C_{12}, C_{16}, $C_{16:1}$, C_{18}, $C_{18:1}$, $C_{18:1}$	C_{12}, C_{14}, C_{15}, C_{16}, $C_{16:1}$,anteiso-C_{17}, C_{17}, C_{18}, $C_{18:1}$	C_{14}, C_{15}, C_{16}, C_{17}, $C_{16:1}$w7c, $C_{18:1}$w7c	C_{16}10-Me, C_{16}, $C_{16:1}$w7, C_{17}cyclo, C_{18}, $C_{18:1}$w7	N.D.	C_{16}, $C_{16:1}$w7, iso-$C_{17:1}$w5
Quinone		-	menaquinone	-	-	
Habitats	deep sea hydrothermal environments	deep sea hydrothermal environments	deep sea hydrothermal environments	hypersaline lakes	hypersaline lakes	hypersaline lakes
References	this study	Takai *et al*., 2009	Mori *et al*., 2011	Sorokin *et al*., 2010	Sorokin *et al*., 2002	Sorokin *et al*., 2007

*: tested as energy and carbon sources

#: utilization of other nitrogen sources were not tested

†; marine strain may tolerate low concentrations of NaCl

N.D.: not determined

References are listed in supplementary text.

(Table S1. continue)

	Chromatiaceae					family *incertae sedis*
Genus	*Thiorhodococcus*	*Allochromatium*	*Marichromatium*	*Thiococcus*	*Thiohalocapsa*	"*Thiobacillus*
Species	*drewsii*	*vinosum*	*gracile*	*pfennigii*	*halophila*	*prosperus*"
Strain	DSM15006[T]					
Cell shape	sphere	rod	rod	sphere	sphere	rod
Cell size (μm)	2.0-3.5	2 x 2.5-6	1.0-1.3 x 2.0-6.0	1.2-1.5	1.5-2.5	0.3 x 3.0-4.0
Motility	+	+	+	-	-	+
Intracellular globules	+	+	+	+	+	+
G+C content (mol %)	64.5 (HPLC)	61.3-66.3 (Bd)/ 64.0 (DSM180 genome, chromosome)	68.9-70.4 (Bd)	69.4-69.9 (Bd)	65.9-66.6 (Tm)	64 (Tm)
Temperature for growth (˚C)						
range	N.D.	N.D.	N.D.	N.D.	N.D.	20-45
optimum	30-35	25-35	20-35	20-35	20-30	33-37
pH for growth						
range	5.2-8.5	6.5-7.6	6.8-7.6	6.5-7.5	6.0-8.0	1-4.5
optimum	6.5-6.7	7.0-7.3	7.2-7.4	7.0	7.0	N.D.
NaCl concentration for growth (%)						
range	0-8	Not required†	N.R.	N.D.	3-20	0-3.5
optimum	2.4-2.6	0	2-3	0.5-2	4-8	0
Chemolithoautotrophy	-	+	+	-	+	+
Electron donors for chemolithotrophy		HS^-, $S_2O_3^{2-}$	HS^-, $S_2O_3^{2-}$		HS^-, $S_2O_3^{2-}$	HS^-, S^0, sulfidic ores
Electron acceptors for chemotrophy		O_2	O_2		O_2	O_2
Phototrophy	+	+	+	+	+	-
Inorganic electron donors for phototrophy	H_2, S^0, HS^-, $S_2O_3^{2-}$	H_2, S^0, HS^-, $S_2O_3^{2-}$, SO_3^{-}	H_2, S^0, HS^-, $S_2O_3^{2-}$, SO_3^{-}	S^0, HS^-	H_2, S^0, HS^-, $S_2O_3^{2-}$, SO_3^{-}	
Mixotrophy	+	+	+	+	+	-
Chemoorganoheterotrophy	-	+	+	-	+	-
Organic carbon sources /organic electron donors						
Formate	+	+	+	-	-	N.D.
Acetate	+	+	+	+	+	N.D.
Pyruvate	+	+	+	+	+	N.D.
Propionate	+	+	+	+	-	N.D.
Butyrate	+	variable	+		-	N.D.
Lactate	-	-	+	+	+	N.D.
Fumarate	+	+	+	+	-	N.D.
Succinate	+	+	+	+	-	N.D.
Citrate	N.D.	-	-	N.D.	-	N.D.
Malate	-	+	+	+	-	N.D.
Fructose	+	-	-	+	+	N.D.
Glucose	-	-	-	-	+	-
Ethanol	+	-	-	-	-	N.D.
Propanol	+	-	N.D.	-	-	N.D.
Glycerol	-	-	-	-	+	N.D.
Yeast Extract	-	N.D.	N.D.	N.D.	+	-
Tryptone peptone	N.D.	N.D.	N.D.	N.D.	N.D.	N.D.
Peptone	-	N.D.	N.D.	N.D.	+	-
Casamino Acids	+	N.D.	+	+	-	N.D.
Gelatin	N.D.	N.D.	N.D.	N.D.	-	N.D.
Nitrogen sources	NH_4^{+} #	N_2, NH_4^+	N_2, NH_4^+	N_2, NH_4^+	NH_4^{+} #	NH_4^{+} #
Major fatty acids	N.D.	C_{14}, C_{16}, $C_{16:1}$, C_{18}, $C_{18:1}$	C_{12}, C_{16}, C_{18}, $C_{16:1}$, $C_{18:1}$w5c	N.D.	N.D.	N.D.
Quinone						
Habitats	salt marsh	sulfidic terrestrial and coastal environments exposed to sunlight	marine sulfidic environments exposed to sunlight	sulfidic terrestrial and coastal environments exposed to sunlight	sulfidic coastal environments exposed to sunlight	coastal hydrothermal environments
References	Zaar *et al*., 2003	Imhoff *et al*., 2005ab Kämpf & Pfennig 1980 Imhoff & Bias-Imhoff 1995	Imhoff *et al*., 2005ac Kämpf & Pfennig 1980 Sucharita *et al*., 2010	Imhoff *et al*., 2005ad	Caumette *et al*., 1991	Huber & Stetter 1989
