# Supplementary material for: Physiological and Genomic Features of a Novel Sulfur-Oxidizing Gammaproteobacterium Belonging to a Previously Uncultivated Symbiotic Lineage Isolated from a Hydrothermal Vent
Source: PLoS One. 2014 Aug 18;9(8):e104959. doi: 10.1371/journal.pone.0104959 (PMC4136832; doi:10.1371/journal.pone.0104959)
Supplement: Text S1 — Additional details on the genomic information of the strain. (DOC) [file pone.0104959.s007.doc]

**Supplementary text S1**

**DNA replication, repair and recombination**

Among genes for DNA replication, each single gene of DnaABGNX, HolABC, LigA, RnhA, PriA, PolA, GyrA, GyrB and TopA and each two genes of DnaEQ, SSB and Rep are present in the genome. Genes of DnaC and HolDE, which load DnaB at *oriC* and are ψ and θ subunits of DNA Pol III, respectively, and a gene of PriB are absent in the genome, as observed in other sulfur-oxidizing *Gammaproteobacteria*. The genome contains genes for DNA repair proteins, such as UvrABCD, Mfd, MutLMSTY, XthA, XseAB and Nth, but those for MutS2, MutH, Phr, Nfo and Ung are absent. The lack of MutH and Nfo is also found in the genomes of other sulfur-oxidizing *Gammaproteobacteria*. A single set of DNA recombination genes including RecAGFJNORQX, AddAB, RuvABC, YqgF, SbcB, RmuC and LexA is encoded, but genes for RecBCD, RecU, Ku (a homologue of YkoV), Lig (a homologue of YkoU) and SbcBC are not found in the genome. RecBCD and AddAB distributed alternatively among *Gammaproteobactria* including symbionts [1], and the deficiency of RecU has been recognized as a common feature of *Gammaproteobacteria* [1,2].

**Protein folding, heat shock proteins and proteins for oxidative stress**

The genome possesses gene sets of heat shock proteins such as sHsp (Hsp15 and Hsp20s), Hsp60 (a set of GroEL and GroS) and Hsp70 (a set of GrpE, DnaK and DnaJ). Genes for heat shock proteases such as HtpX, HslV, Lon and Clp, and those for protein folding, such as thioredoxin, glutaredoxin and peptidyl-prolyl cis-trans isomerase, are present. Among representative proteins for oxidative stress, those of superoxide dismutase, thiol peroxidase, cytochrome *c* peroxidase and alkyl hydroperoxide reductase are encoded, but those of catalase and glutathione peroxidase are missing from the genome.

**Motility, sensor, chemotaxis and signaling**

An almost complete gene set of flagellar biosynthesis proteins and flagellar proteins is concentrated in one genomic region with at least 5 gene clusters; *fliKLMNOPQR-flhBAFG-fliA*, *flrBC-fliEFGHIJ*, *flgBCDEFGHIJKL-fliC1C2-flaG-fliDS-*(TBH_C1378), *flgAMN-*(TBH_C1358)-*motAB-*(TBH_C1355)-*pilZ,* and *flaK.* The strain has two flagellin proteins, FliC1 and C2. Negative regulator flgM and flagellar chaperon/transcriptional regulator FlgN have only been found in *Betaproteobacteria* and enteric bacteria [3,4]. Genes for flagellar switch phosphatase FliY, transcriptional activators FliZ and FlhCD and flagellar protein FlhE [3-8] are absent. An apparent homologue of the flagellar chaperon/transcriptional regulator FliT was not identified, but a hypothetical protein (TBH_C1378) harbors FliT domain. A gene of hypothetical protein (TBH_C1355) shows low similarity with *pilZ.* FliATY have been found only in enteric bacteria and other specific groups [3,4]. The anomalously concentrated localization of flagella-related gene clusters in the genome of strain Hiromi 1 compared with other genomes may reflect the absence of certain transcriptional regulators. Thus, the lack of these proteins may not affect flagellar formation and function in this organism.

No flagella formation was observed in sulfur-oxidizing gammaproteobacterial endosymbionts, including *I. nautilei* endosymbionts [9]. In addition, the endosymbiotic state of “*Ca*. E. persephone*”,* an endosymbiont of *Riftia* tubeworms and scaly-foot gastropods, lacks flagella but also harbors the complete set of flagella-related genes, and the ability to form flagella has been predicted in metagenomic analyses [10,11]. Considering the lifestyle of the strain Hiromi 1, which prefers to grow on elemental sulfur with biofilm, the flagellum may be not expressed at the adhesive lifestyle.

An almost complete gene set of type IV pilus formation is scattered throughout the genome, with 10 locations: *yfgB-pilF-yfgA*, *pilH*, *ispG*, *pilA*, *pilBCD-coaE*-*yacG*, *pilMNOPQ*, *ponA*, *yggS*, *pilTU*, *pilGHIJY, pilSR* and *ispH-fimTU-pilVWXYE* [12]. Methyltransferase PilK and pilus assembly protein PilX are absent.

In addition to the two-component signal transduction systems (TCSs) described in the main text, such as the homologues of QseB/QseC, QseE/QseF, RpfC/RpfG, TCSs homologous to PhoB/PhoR, PhoP/PhoQ, BarA/UvrY, EnvZ/OmpR, RstA/RstB, CbrA/CbrB, AlgZ/AlgR and NarL/NarX that may relate to virulence traits were identified in the genome while capability of virulence has not been observed in this strain. The PhoB/PhoR system found in diverse bacteria controls phosphate regulon plays a key role in phosphate homeostasis. In pathogens, the system also affects the expression of virulence traits [13]. The PhoP/PhoQ system senses Mg^2+^ concentrations as a specific signal and regulates functional genes, including metal uptake and virulence genes. The RstA/RstB system found in the genome of the isolate is considered to work for iron uptake with the PhoP/PhoQ system [14,15], but the PmrA/PmrB system that is activated by PhoP/PhoQ was not detected in the genome [14,16]. The BarA/UvrY system, of which genes are detected in the genome, is activated by weak acids and TCA cycle intermediates and indirectly regulates various functions, such as carbon metabolism, motility, virulence, quorum sensing, biofilm formation and oxidative stress through non-coding RNAs and CsrA [17]. The EnvZ/OmpR system was originally found as a response regulator for osmolality changes but is now recognized as a global regulator with roles in amino acid metabolism, transport, flagellar synthesis and virulence [17-19]. The global regulator CbrA/CbrB found in *Pseudomonas aeruginosa* modulates catabolic pathways involved in carbon and nitrogen utilization [20]. The TCS also regulates virulence-related functions through small RNAs and Crc, and CbrA itself also modulates the expression of PhoPQ and other TCSs [21]. The TCS of AlgZ/AlgR regulates not only alginate biosynthesis but also virulence factors including twitching motility, hydrogen cyanide production and biofilm formation in *P. aeruginosa* [22]. As in the case of *P. aeruginosa*, *algH* is juxtaposed with the *algZR* operon on the reverse strand [22]. The NarL/NarX system senses oxygen, nitrate and nitrite and regulates anaerobic respiration [23]. Moreover, the genome also harbors 12 gene clusters of TCSs and three orphan histidine kinase genes. The GGDEF and/or EAL domains that correspond with diguanylate cyclase and phosphodiesterase activity, respectively, and regulate biofilm formation, autoaggregation and motility [24], occur in seven CDSs. Among these sensors, PAS domains, which are ubiquitous and monitor environmental changes in light, redox potential, O_2_, small ligands, and the overall energy level of the cell [25], are found in five histidine kinases and one GGDEF/EAL domain protein.

Gene for transcriptional regulators for respiration and oxidative stress response found in *Gammaproteobacteria*, such as Fnr, DusB/Fis, OxyR and Fur are present. However, ArcA and CpxR/CpxA genes are absent in the genome [26].

Transcription of 12 sensor genes that may relate to virulence and chemotaxis was verified by RT-PCR analysis using cells grown under free-living conditions in both chemolithoautotrophic and chemolithomixotrophic media. The sensor genes examined were NtrC family TCS (TBH_C0328/0329), RpfC/RpfG (TBH_C1049/1051), RpfF (TBH_C1052), CbrA (TBH_C1065/1066), BarA/UvrY (TBH_C1133/0870), FlrB/FlrC (TBH_C1380/1381), WspE (TBH_C1475), QseB/QseC (TBH_C1833/1834), QseB/QseC (TBH_C2290/2291), CheA (TBH_C2123), QseE/QseF (TBH_C2638/2640) and RstA/RstB (TBH_C2787/2788). As a result, the transcription of genes for CbrA/CbrB, BarA/UvrY, FlrB/FlrC, WspE and QseB/QseC (TBH_C1833/1834) were not detected, and the presence of organic compounds did not regulate transcription. Although it seems most likely that these genes can be transcribed only in specific living states or under specific growth conditions, the results suggest that the transcription of these sensor genes may be suppressed under normal growth conditions, even under chemolithoautotrophic and chemolithoheterotrophic growth conditions.

**Transporters and secretion system**

For the potential adaptation to the metal-rich environments of the habitats, the strain has various gene components for a heavy metal efflux system, including an ACR3 family arsenite transporter, a mercuric ion transporting system, two Cu^2+^-exporting ATPases, two Fe^2+^/Pb^2+^ transport systems, a tellurite resistance/dicarboxylate transporter, an arsenical pump family protein, an Fe^3+^ transport protein, an Fe^2+^ transport protein, peptide/nickel transport systems, a Mn^2+^/Zn^2+^ ABC transporter, a Mg^2+^/Co^2+^ transporter, a molybdenum ABC transporter and a metal ion ABC transporter. Genes for an iron-uptaking FepCD iron complex transporter and two ExbBD-TonB systems [27] and eight resistance-nodulation-cell division super family (RND)-type transporters and one cation diffusion facilitator family (CDF) transporter of unknown function are also encoded in the genome. Interestingly, some of these transporters such as the ACR3 arsenite transporter, the CDF family cation diffusion facilitator, the RND family efflux transporter, the arsenical pump family proteins, the PiT family inorganic phosphate transporter, and the ABC transporter permease, did not show significant amino acid sequence similarity with gammaproteobacterial entities. However, they showed significant similarity with sequences found in genomes of other proteobacterial classes, *Firmicutes* and/or deeply branching *Bacteria* and *Archaea* including species that have been observed in hydrothermal environments.

The presence of genes for the outer-membrane protein TolC and several HlyD-like proteins with an MPF domain, which are parts of an ABC transporter, a continuous channel and an outer membrane protein structure [28], suggests the possible operation of a type I protein secretion system in the strain. Genes for inner membrane transporting systems Sec (SecABDEF/YidC) and Tat (TatABC) for unfolded and folded proteins, respectively [29,30] are present. An incomplete gene cluster of type II secretion pathway *gspEFGHIJKLMND* without *gspCO* is encoded in the genome. A similar gene organizations, in which *gspD* is placed downstream of *gspN* but not upstream of *gspE* and in which *gspCO* is absent, is also found in the genomes of *Nitrosococcus* species [31]. Thus, the GspCO might not be absolutely necessary for the function of the secretion system. Apparent homologues of previously characterized type IV secretion system transporters that also function with Sec and Tat system [32] have not yet been identified in the genome. Genes for type III protein secretion injectisome, type IV DNA protein secretion system, and type VI secretion system (T6SS) [33-35] are absent in this genome. Genes for lipoprotein-releasing ABC transporter LolCD complex and periplasmic chaperon LolA [36] were observed, while apparent homologue of LolB and LolE (the orthologous subunit of LolC) are absent.

Genes for a lipopolysaccharide export system, which consists of LptABCDEFG and MsbA [37], and an outer membrane phospholipid-importing system of MlaACDEF [38] are present, while an MlaB subunit gene is missing. A gene cluster of the TolABQR-PAL system (*ybgC-tolQRAB-pal-ybgF*) is encoded in the genome. Two gene sets of Wzm/Wzt-like ABC transporters that may be responsible for lipopolysaccharide transport [39] were identified.

The genome harbors genes of two amino acid/polyamine transporters (APC family) and genes for a glutamate permease (ESS family) and an oligopeptide transporter, while those of sugar transporters (TRAP-T family) are not found. The presence of these transporters is consistent with the utilization of organic acids and proteinaceous compounds as carbon sources by strain Hiromi 1.

**References in supplementary information**

1. 1. Cromie GA (2009) Phylogenetic ubiquity and shuffling of the bacterial RecBCD and AddAB recombination complexes. J Bacteriol 191: 5076-5084.
2. 2. Rocha EP, Cornet E, Michel B (2005) Comparative and evolutionary analysis of the bacterial homologous recombination systems. PLoS Genet 1: e15.
3. 3. Chilcott GS, Hughes KT (2000) Coupling of flagellar gene expression to flagellar assembly in *Salmonella enterica* serovar *typhimurium* and *Escherichia coli*. Microbiol Mol Biol Rev 64: 694-708.
4. 4. Liu R, Ochman H (2007) Origins of flagellar gene operons and secondary flagellar systems. J Bacteriol 189: 7098-7104.
5. 5. Szurmant H, Ordal GW (2004) Diversity in chemotaxis mechanisms among the bacteria and archaea. Microbiol Mol Biol Rev 68: 301-319.
6. 6. Yamamoto S, Kutsukake K (2006) FliT acts as an anti-FlhD2C2 factor in the transcriptional control of the flagellar regulon in *Salmonella enterica* serovar typhimurium. J Bacteriol 188: 6703-6708.
7. 7. Hirano T, Mizuno S, Aizawa S, Hughes KT (2009) Mutations in *flk*, *flgG*, *flhA*, and *flhE* that affect the flagellar type III secretion specificity switch in *Salmonella enterica*. J Bacteriol 191: 3938-3949.
8. 8. Saini S, Koirala S, Floess E, Mears PJ, Chemla, YR, et al. (2010) FliZ induces a kinetic switch in flagellar gene expression. J Bacteriol 192: 6477-6481.
9. 9. Windoffer R, Giere O (1997) Symbiosis of the hydrothermal vent gastropod *Ifremeria nautilei* (*Provannidae*) with endobacteria - Structural analyses and ecological considerations. Biol Bull 193: 381-392.
10. 10. Robidart JC, Bench SR, Feldman RA, Novoradovsky A, Podell SB, et al. (2008) Metabolic versatility of the *Riftia pachyptila* endosymbiont revealed through metagenomics. Environ Microbiol 10: 727-737.
11. 11. Nakagawa S, Shimamura S, Takaki Y, Suzuki Y, Murakami SI, et al. (2014) Allying with armored snails: the complete genome of gammaproteobacterial endosymbiont. ISME J 8: 40-51.
12. 12. Pelicic V (2008) Type IV pili: e pluribus unum? Mol Microbiol 68: 827-837.
13. 13. Lamarche MG, Wanner BL, Crépin S, Harel J (2008) The phosphate regulon and bacterial virulence: a regulatory network connecting phosphate homeostasis and pathogenesis. FEMS Microbiol Rev 32: 461-473.
14. 14. Choi E, Groisman EA, Shin D (2009) Activated by different signals, the PhoP/PhoQ two-component system differentially regulates metal uptake. J Bacteriol 191: 7174-7181.
15. 15. Nam D, Choi E, Kweon DH, Shin D (2010) The RstB sensor acts on the PhoQ sensor to control expression of PhoP-regulated genes. Mol Cells 30: 363-368.
16. 16. Groisman EA (2001) The pleiotropic two-component regulatory system PhoP-PhoQ. J Bacteriol 183: 1835-1842.
17. 17. Beier D, Gross R (2006) Regulation of bacterial virulence by two-component systems. Curr Opin Microbiol 9: 143-152.
18. 18. Oshima T, Aiba H, Masuda Y, Kanaya S, Sugiura M, et al. (2002) Transcriptome analysis of all two-component regulatory system mutants of *Escherichia coli* K-12. Mol Microbiol 46: 281-291.
19. 19. Calva E, Oropeza R (2006) Two-component signal transduction systems, environmental signals, and virulence. Microb Ecol 51: 166-176.
20. 20. Li W, Lu CD (2007) Regulation of carbon and nitrogen utilization by CbrAB and NtrBC two-component systems in *Pseudomonas aeruginosa*. J Bacteriol 189: 5413-542.
21. 21. Yeung AT, Bains M, Hancock RE (2011) The sensor kinase CbrA is a global regulator that modulates metabolism, virulence and antibiotic resistance in *Pseudomonas aeruginosa*. J Bacteriol 193: 918-31.
22. 22. Cody WL, Pritchett CL, Jones AK, Carterson AJ, Jackson D, et al. (2009) *Pseudomonas aeruginosa* AlgR controls cyanide production in an AlgZ-dependent manner. J Bacteriol 191: 2993-3002.
23. 23. Stewart V, Chen LL, Wu HC (2003) Response to culture aeration mediated by the nitrate and nitrite sensor NarQ of *Escherichia coli* K-12*.* Mol Microbiol 50: 1391-1399.
24. 24. Römling U, Gomelsky M, Galperin MY (2005) C-di-GMP: the dawning of a novel bacterial signalling system. Mol Microbiol 57: 629–639.
25. 25. Taylor BL, Zhulin IB (1999) PAS domains: internal sensors of oxygen, redox potential, and light. Microbiol Mol Biol Rev 63: 479-506.
26. 26. Ravcheev DA, Gerasimova AV, Mironov AA, Gelfand MS (2007) Comparative genomic analysis of regulation of anaerobic respiration in ten genomes from three families of gamma-proteobacteria (*Enterobacteriaceae*, *Pasteurellaceae*, *Vibrionaceae*). BMC Genomics 8: 54.
27. 27. Chu BC, Garcia-Herrero A, Johanson TH, Krewulak KD, Lau CK, et al. (2010) Siderophore uptake in bacteria and the battle for iron with the host; a bird's eye view. Biometals 23: 601-611.
28. 28. Holland IB, Schmitt L, Young J (2005) Type 1 protein secretion in bacteria, the ABC-transporter dependent pathway (review). Mol Membr Biol 22: 29-39.
29. 29. De Buck E, Lammertyn E, Anné J (2008) The importance of the twin-arginine translocation pathway for bacterial virulence. Trends Microbiol 16: 442-453.
30. 30. Mori H, Ito K (2001) The Sec protein-translocation pathway. Trends Microbiol 9: 494-500.
31. 31. Evans FF, Egan S, Kjelleberg S (2008) Ecology of type II secretion in marine *gammaproteobacteria*. Environ Microbiol 10:1101-1107.
32. 32. Henderson IR, Navarro-Garcia F, Desvaux M, Fernandez RC, Ala'Aldeen D (2004) Type V protein secretion pathway: the autotransporter story. Microbiol Mol Biol Rev 68: 692-744.
33. 33. Cornelis GR (2006) The type III secretion injectisome. Nat Rev Microbiol 4: 811-825.
34. 34. Alvarez-Martinez CE, Christie PJ (2009) Biological diversity of prokaryotic type IV secretion systems. Microbiol Mol Biol Rev 73: 775-808.
35. 35. Boyer F, Fichant G, Berthod J, Vandenbrouck Y, Attree I (2009) Dissecting the bacterial type VI secretion system by a genome wide in silico analysis: what can be learned from available microbial genomic resources? BMC Genomics 10: 104.
36. 36. Tokuda H (2009) Biogenesis of outer membranes in Gram-negative bacteria. Biosci Biotechnol Biochem 73: 465-473.
37. 37. Chng SS, Ruiz N, Chimalakonda G, Silhavy TJ, Kahne D (2010) Characterization of the two-protein complex in Escherichia coli responsible for lipopolysaccharide assembly at the outer membrane. Proc Natl Acad Sci USA 107: 5363-5368.
38. 38. Malinverni JC, Silhavy TJ (2009) An ABC transport system that maintains lipid asymmetry in the gram-negative outer membrane. Proc Natl Acad Sci USA 106: 8009-8014.
39. 39. Rocchetta HL, Lam JS (1997) Identification and functional characterization of an ABC transport system involved in polysaccharide export of A-band lipopolysaccharide in *Pseudomonas aeruginosa*. J Bacteriol 179: 4713-472.
40. 40. Mori K, Suzuki K, Urabe T, Sugihara M, Tanaka K, et al. (2011) *Thioprofundum hispidum* sp. nov., an obligately chemolithoautotrophic sulfur-oxidizing bacterium of class *Gammaproteobacteria* isolated from the hydrothermal field in Suiyo Seamount, and proposal of *Thioalkalispiraceae* fam. nov. in the order of *Chromatiales*. Int J Syst Evol Microbiol 61: 2412-2418.
41. 41. Sorokin DY, Kovaleva OL, Tourova TP, Muyzer G (2010) *Thiohalobacter thiocyanaticus* gen. nov., sp. nov., a moderately halophilic, sulfur-oxidizing gammaproteobacterium from hypersaline lakes, that utilizes thiocyanate. Int J Syst Evol Microbiol 60: 444-450.
42. 42. Sorokin DY, Tourova TP, Kolganova TV, Sjollema KA, Kuenen JG (2002) *Thioalkalispira microaerophila* gen. nov., sp. nov., a novel lithoautotrophic, sulfur-oxidizing bacterium from a soda lake. Int J Syst Evol Microbiol 52: 2175-2182.
43. 43. Sorokin DY, Tourova TP, Bezsoudnova EY, Pol A, Muyzer G (2007) Denitrification in a binary culture and thiocyanate metabolism in *Thiohalophilus thiocyanoxidans* gen. nov. sp. nov. a moderately halophilic chemolithoautotrophic sulfur-oxidizing gammaproteobacterium from hypersaline lakes. Arch Microbiol 187: 441-450.
44. 44. Zaar A, Fuchs G, Golecki JR, Overmann J (2003) A new purple sulfur bacterium isolated from a littoral microbial mat, *Thiorhodococcus drewsii* sp. nov. Arch Microbiol 179: 174-183.
45. 45. Imhoff JF (2005a) Family I. *Chromatiaceae* Bavendamm 1924, 125AL emend. Imhoff 1984b, 339. In: Brenner DJ, Krieg NR, Staley JT, Garrity GM, editors. Bergey’s Manual of Systematic Bacteriology, 2nd edn, vol. 2, part B. New York, USA: Springer. pp. 3–9.
46. 46. Imhoff JF (2005b) Genus II. *Allochromatium* Imhoff, Süling and Petri 1998b, 1140VP. In: Brenner DJ, Krieg NR, Staley JT, Garrity GM, editors. Bergey’s Manual of Systematic Bacteriology, 2nd edn, vol. 2, part B. New York, USA: Springer. pp. 12–14.
47. 47. Kämpf C, Pfennig N (1980) Capacity of *Chromatiaceae* for chemotrophic growth. Specific respiration rates of *Thiocystis violacea* and *Chromatium vinosum*. Arch Microbiol 127: 125-135.
48. 48. Imhoff JF, Bias-Imhoff U (1995) Lipids, quinones and fatty acids of anoxygenic phototrophic bacteria. In: Blankenship RE, Madigan MT, Bauer CE, editors. Anoxygenic phototrophic bacteria. Dordrecht, Netherlands: Kluwer Academic Publishers. pp. 179-205.
49. 49. Imhoff JF (2005c) Genus VII. *Marichromatium* Imhoff, Süling and Petri 1998b, 1140VP. In: Brenner DJ, Krieg NR, Staley JT, Garrity GM, editors. Bergey’s Manual of Systematic Bacteriology, 2nd edn, vol. 2, part B. New York, USA: Springer. pp. 20–21.
50. 50. Sucharita K, Shiva Kumar E, Sasikala Ch, Panda BB, Takaichi S, Ramana ChV (2010) *Marichromatium fluminis* sp. nov., a slightly alkaliphilic, phototrophic gammaproteobacterium isolated from river sediment. Int J Syst Evol Microbiol 60: 1103-1107.
51. 51. Imhoff JF (2005d) Genus VII. *Thiococcus* Imhoff, Süling and Petri 1998b, 1139VP. In: Brenner DJ, Krieg NR, Staley JT, Garrity GM, editors. Bergey’s Manual of Systematic Bacteriology, 2nd edn, vol. 2, part B. New York, USA: Springer. pp. 28–29.
52. 52. Caumette P, Baulaigue R, Matheron R (1991) *Thiocapsa halophila* sp. nov., a new halophilic phototrophic purple sulfur bacterium. Arch Microbiol 155: 170-176.
53. 53. Huber H, Stetter KO (1989) *Thiobacillus prosperus* sp. nov., represents a new group of halotolerant metal-mobilizing bacteria isolated from a marine geothermal field. Arch Microbiol 151: 479-485.
